# Printable, Highly Sensitive Flexible Temperature Sensors for Human Body Temperature Monitoring: A Review

**DOI:** 10.1186/s11671-020-03428-4

**Published:** 2020-10-15

**Authors:** Yi Su, Chunsheng Ma, Jing Chen, Huiping Wu, Weixiang Luo, Yueming Peng, Zebang Luo, Lin Li, Yongsong Tan, Olatunji Mumini Omisore, Zhengfang Zhu, Lei Wang, Hui Li

**Affiliations:** 1 College of Mechanical Engineering, North University of China, Taiyuan, 030051 Shanxi China; 2 Institute of Biomedical and Health Engineering, Shenzhen Institutes of Advanced Technology, Chinese Academy of Sciences, Shenzhen, 518055 Guangdong China; 3 Nursing Department, Shenzhen People’s Hospital (The Second Clinical Medical College, Jinan University; The First Affiliated Hospital, Southern University of Science and Technology), Shenzhen, 518020 Guangdong China; 4 Neonatal Intensive Unit, Shenzhen People’s Hospital (The Second Clinical Medical College, Jinan University; The First Affiliated Hospital, Southern University of Science and Technology), Shenzhen, 518020 Guangdong China

**Keywords:** Flexible sensor, Temperature sensor, Body temperature monitoring, Printable sensor, Wearable electronics

## Abstract

In recent years, the development and research of flexible sensors have gradually deepened, and the performance of wearable, flexible devices for monitoring body temperature has also improved. For the human body, body temperature changes reflect much information about human health, and abnormal body temperature changes usually indicate poor health. Although body temperature is independent of the environment, the body surface temperature is easily affected by the surrounding environment, bringing challenges to body temperature monitoring equipment. To achieve real-time and sensitive detection of various parts temperature of the human body, researchers have developed many different types of high-sensitivity flexible temperature sensors, perfecting the function of electronic skin, and also proposed many practical applications. This article reviews the current research status of highly sensitive patterned flexible temperature sensors used to monitor body temperature changes. First, commonly used substrates and active materials for flexible temperature sensors have been summarized. Second, patterned fabricating methods and processes of flexible temperature sensors are introduced. Then, flexible temperature sensing performance are comprehensively discussed, including temperature measurement range, sensitivity, response time, temperature resolution. Finally, the application of flexible temperature sensors based on highly delicate patterning are demonstrated, and the future challenges of flexible temperature sensors have prospected.

## Introduction

All life activities of the human body based on metabolism and relatively constant body temperature is necessary for a healthy metabolism [1]. Hyperthermia or hypothermia will affect the activity of enzymes in the body, thereby affecting the regular operation of human metabolism, causing disorders of various cells, tissues and organs, and even death in severe cases. It can see that body temperature's relative stability is a necessary condition for maintaining a stable environment in the body and ensuring the regular progress of life activities such as metabolism. For the human body, changes in body temperature reflect much information about human health, and abnormal changes in body temperature usually indicate poor health. In human health monitoring [2, 3], body temperature is an essential factor that cannot ignore, and real-time and accurate monitoring of body temperature is particularly important.

Flexible temperature sensors have been developing towards wearable, highly sensitive, portable, large-area, accurate, and real-time trends. The flexible temperature sensor mainly uses the electrical signal change of the thermosensitive material due to the temperature change to realize the real-time monitoring of the temperature [4]. It also uses the character of the flexible substrate to adhere strictly to the skin to realize its function. Compared with traditional temperature measuring instruments, in addition to being difficult to carry, expensive, and applicable to monitoring occasions, there are limitations, due to the intentional or unintentional movement of the patient and the inability to monitor specific locations (such as wounds [5], tumor ablation sites in the body [6]), can easily lead to inaccurate or imperfect measurement results. In order to solve the above problems, wearable flexible, thin and sensitive patterned temperature sensors have become a research hotspot for scientific researchers.

In recent years, the research of flexible temperature sensors for monitoring body temperature has continuously developed, and there are many innovations [7]. The use of patterned fabricating to achieve large-area fabricating of flexible temperature sensors has become a development trend [8]; imitating biological structures in nature is an excellent idea [9]. The octopus feet with adsorption properties, the viper's cheeks that can sense changes in biological temperature [10], and the whisker-like structures [11] of some arthropods or mammals also have a temperature sensing function; in order to clearly display the body temperature monitored by the sensor, the researchers will the flexible temperature sensor is arrayed [12–14], and the imaging device or its electrochromic material can use to visualize the thermal imaging mapping [5, 15, 16]. In-depth research on flexible temperature sensors also provides appropriate technical support for meeting high requirements such as high-sensitivity monitoring of body temperature.

This article reviews the recent research progress of high-sensitivity flexible temperature sensors in human body temperature monitoring, heat-sensitive materials, manufacturing strategies, basic performance, and applications. The first part will select materials for flexible temperature sensors and summarize a variety of flexible substrates, heat-sensitive materials that can be used as flexible temperature monitoring sensors. The second part focuses on the use of flexible temperature sensors in the literature in recent years. The patterned manufacturing method is reviewed, showing the typical manufacturing process. The third part introduces the critical performance parameters of temperature sensors. The fourth part shows the application scenarios and practical applications of flexible temperature sensors in recent years. Finally, the potential challenges and future development prospects of printable, highly sensitive flexible temperature sensors are briefly discussed.

## Methods

### Material

#### Flexible Substrates

In recent years, the application and research of flexible materials in the fields of electronic technology and medical and health has gradually increased. The fabricate of flexible sensors requires the sensor itself to be flexible, stretchable, and ductile and the substrates and circuits on which it depends. Specific stretch and stretch characteristics to adapt to the adhesion on the human body surface, common flexible substrates are usually processed into a film, such as polydimethylsiloxane (PDMS) [17–20], polyimide (PI) [21, 22], polyurethane (PU) [23], polyethylene terephthalate (PET) [24, 25], polyvinyl alcohol(PVA) [26], polyvinyl butyral(PVB) [27], paper [28, 29], silicone rubber [5, 30, 31], and more skin-friendly biodegradable materials can also be used, such as pectin [32], cotton, silk [33], and other cellulose materials [34, 35].

**Fig. 1:**
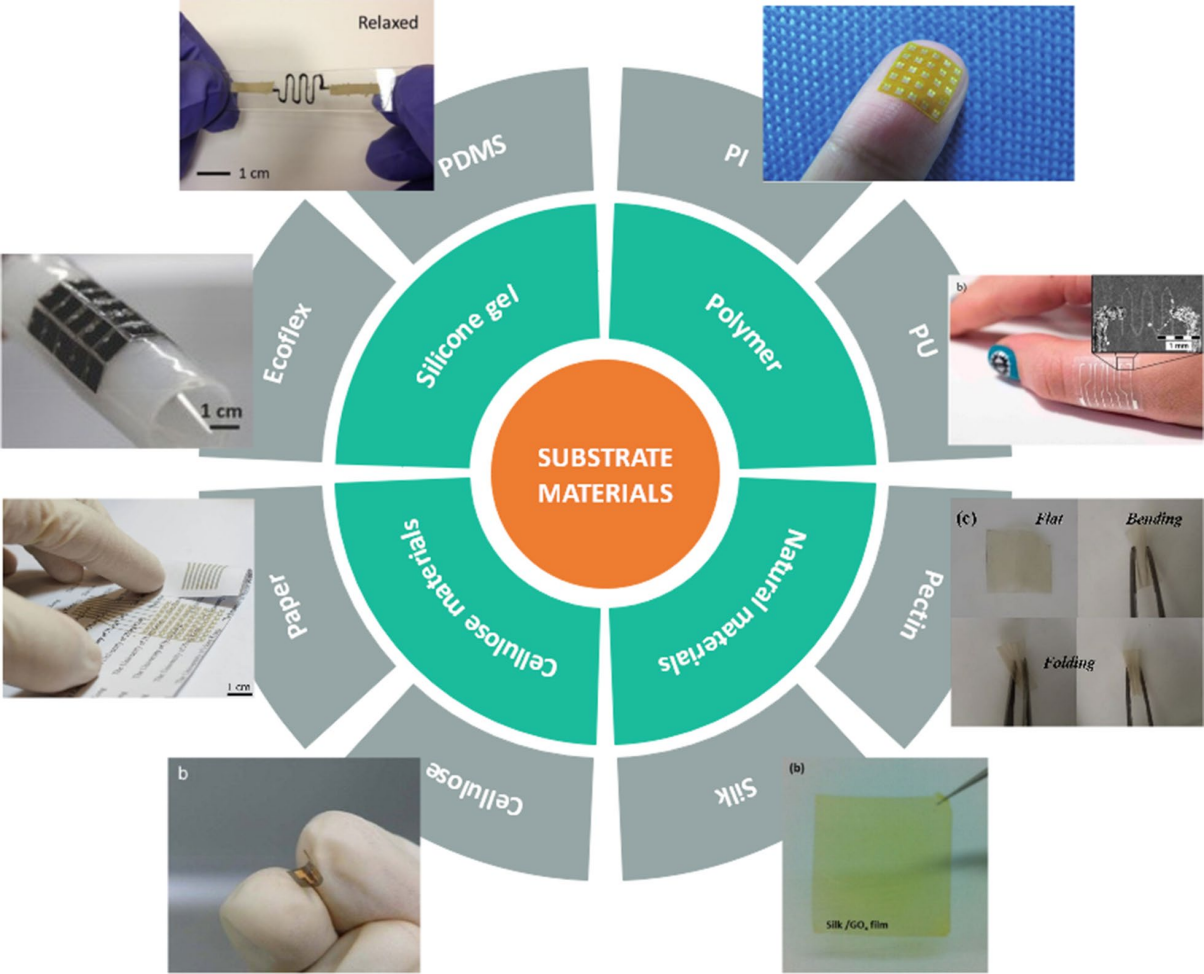
Schematic illustration of the substrate materials for part of flexible sensors. Clockwise from right top: polyimide (PI) [55], polyurethane (PU) [56], pectin [32], silk [33], cellulose [57], paper [28], ecoflex [31] polydimethylsiloxane (PDMS) [58]

At present, the most used flexible substrate in flexible temperature sensors is PDMS, which is an excellent thermal and electrical insulating material, with a relative permittivity of 2.3–2.8 and a volume resistivity of ${1}{\text{.2}} \times {10}^{{14 }} \,\Omega \,{\text{cm}}^{ - 1}$, its specific gravity is $1.03\,{\text{kg}}\,{\text{m}}^{ - 3}$ at 25 °C, PDMS has better thermal stability, and the thermal conductivity of PDMS is $0.15\,{\text{W}}\,{\text{m}}^{ - 1} \,{\text{K}}^{ - 1}$.[36] The glass transition temperature is as low as 125 °C, the coefficient of thermal expansion (CTE) [37] is $301\,{\text{ppm}}\,^{ \circ } {\text{C}}^{ - 1}$. And it's Young's modulus is $\approx 3.7 \,{\text{MPa}}$ [38], making the stretch and strain more than 200%. Given its excellent stretch, extensibility, thermoelectric properties [39–41], high chemical stability, and easy use make PDMS more abundant in applications such as electronic skin [42]. Polyimide (PI) material with properties similar to PDMS (shown in Table 1), PI exhibit a poor thermal conductivity in the order of $0.1\,{\text{W}}\,{\text{m}}^{ - 1} \,{\text{K}}^{ - 1}$ [43–45], with electrical insulating resistivity ${1}{\text{.5}} \times {10}^{{{17}}} \, \Omega \,{\text{cm}}^{ - 1}$ [46]. The relative dielectric constant 3.0–3.6, with a higher glass transition temperature (360–410 °C) [47] and a lower coefficient of thermal expansion (CTE) ($16\,{\text{ppm}}\,^{ \circ } {\text{C}}^{ - 1}$). It is Young's modulus ≈$2.8\,{\text{GPa}}$ [6, 48]. Polyurethane (PU) materials with bio-adaptability, good stretch ductility [49], economical, and practical use in temperature sensors for human body temperature monitoring [50]. The thin-film flexible substrate not only has excellent mechanical properties, but also is suitable for application research in the field of flexible temperature sensor based on excellent thermal properties. As shown in Fig. 1. In addition to using the aforementioned organic polymer materials in flexible sensing, common fabrics or other biodegradable materials, such as fabric [51, 52], silk [53] and cotton [54], are also soft and deformable, lightweight, economical, breathable, comfortable, durable, and reusable. Other advantages, it is also expected and concerned to be studied as a base material for flexible temperature sensors.

**Table 1:** Contrast of common flexible substrates

Flexible substrates	Thermal conductivity	CTE	Young's modulus	Glass transition temperature	Resistivity	Relative permittivity
PDMS	$0.15{ }\,{\text{W}}\,{\text{m}}^{ - 1} \,{\text{K}}^{ - 1}$	$301\,{\text{ppm}}\,^{ \circ } {\text{C}}^{ - 1}$	$\approx \,3.7\,{\text{MPa}}$	125 $^{ \circ } {\text{C}}$	${1}{\text{.2}} \times {10}^{{14 }} \,\Omega \,{\text{cm}}^{ - 1}$	2.3–2.8
PI	$0.1\,{\text{W}}\,{\text{m}}^{ - 1} \,{\text{K}}^{ - 1}$	$16{ }\,{\text{ppm}}\,^{ \circ } {\text{C}}^{ - 1}$	$\approx \,2.8 \,{\text{GPa}}$	360–410 $^{ \circ } {\text{C}}$	${1}{\text{.5}} \times {10}^{{{17}}} \, \Omega \,{\text{cm}}^{ - 1}$	3.0–3.6

#### Thermal Material

The active material is sensitive in the sensor that is responsible for directly and effectively responding to heat sources and thermal signals, its properties directly determine the performance of the temperature sensor, including the sensitivity to temperature, the length of response time, the durability, and the resolution to temperature [59]. A heat-sensitive material that is simple to fabricate, reasonable in raw materials, bio-adaptable, and has a certain degree of ductility and excellent performance has greater appeal for in-depth research on flexible temperature sensors [60–62].

##### Carbon

Much effort has made to create temperature-sensitive conductive composite materials by incorporating different conductive fillers (such as carbon-based materials, conductive polymers, and metal particles) into semiconductors and insulating polymers matrices. Common carbon materials include carbon black (CB), graphite (Gr), carbon nanotubes (CNTs), and graphene.

**Fig. 2:**
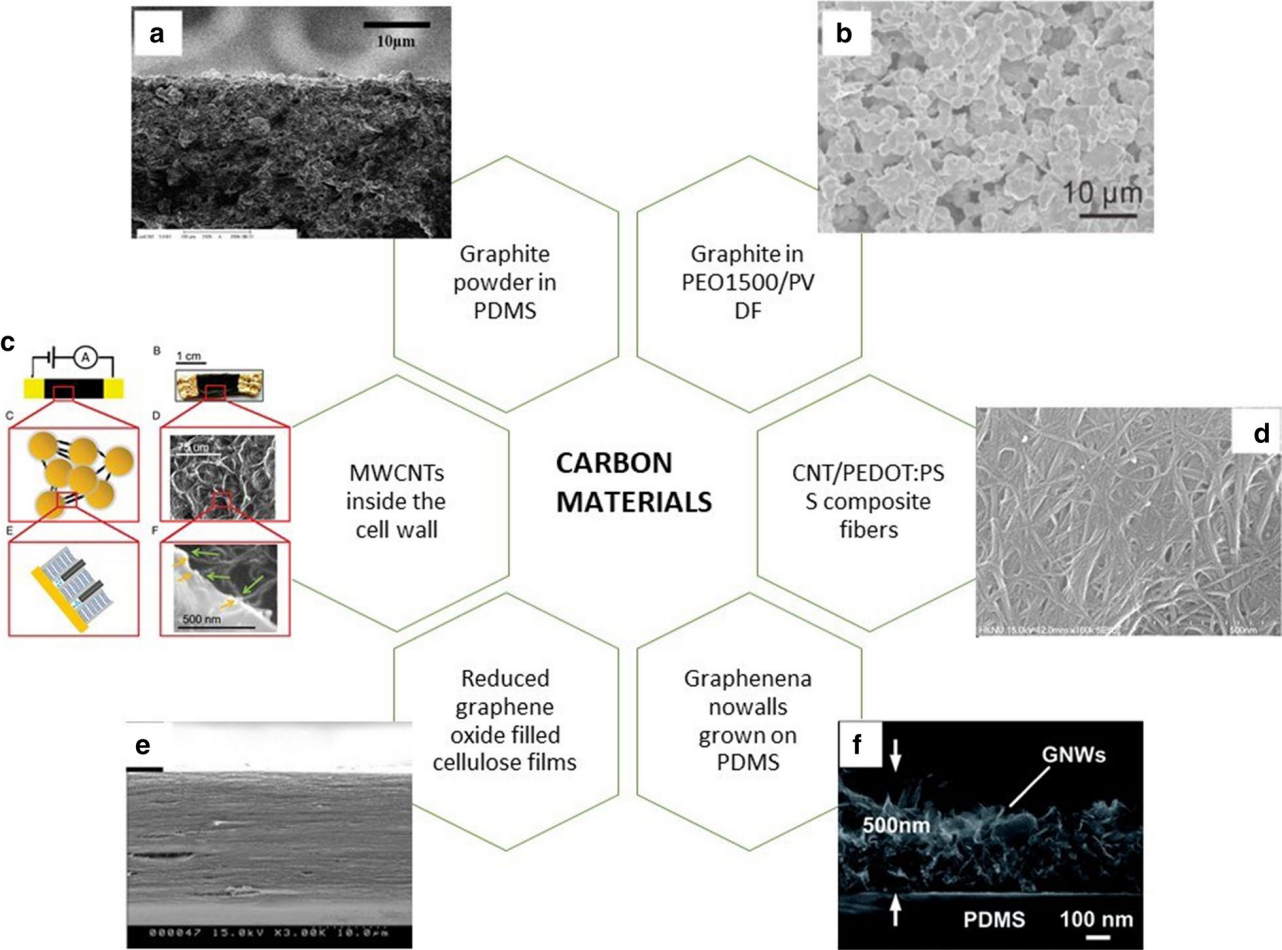
Various flexible temperature sensors based on carbon materials. **a** SEM images of a graphite-PDMS composite [67]. **b** The entire surface micrographs of PEO1500/PVDF/Gr [68]. **c** Schematic diagrams and scanning electron microscopy (SEM) images of cyberwood [76]. **d** SEM images of the CNT/PEDOT:PSS composite fibers with CNT contents of 40 wt% [75]. **e** SEM images of CrGO100 [54]. **f** The cross-section SEM image of GNWs on PDMS [86]

Carbon black (CB) and graphite often used as conductive fillers due to their excellent electronic and mechanical properties and low processing costs. Among them, CB is easy to form aggregates when mixing with polymers to form composite materials, and temperature changes will affect its electrical properties [63]. Stability results in a higher temperature coefficient of resistance (TCR) [64]. Graphite is an allotrope of carbon, with good electrical conductivity, thermal conductivity and chemical stability, and a thermal expansion coefficient less at $5.0 \times 10^{ - 6} \,{\text{K}}^{ - 1}$ [65]. Compared with carbon black, graphite powder as a conductive filler is more sensitive to temperature, and the two and their mixtures will reduce the percolation threshold of the temperature-sensitive material composite film formed by the filled polymer. Expanded graphite (EG) is a new type of functional carbon material that loose and porous worm-like substance obtained from natural graphite flakes through intercalation, washing, drying, and high-temperature expansion. The thermal conductivity of the composite formed by hot pressing after the material can reach $4.70\,{\text{W}}\,{\text{m}}^{ - 1} \,{\text{K}}^{ - 1} { }$ when 10 wt% of EG is impregnated into PDMS prepolymer [66]. Shih et al. [67]used PDMS as the base material and graphite powder as the heat-sensitive sensing material, and used the PI film through the printing process produced a flexible temperature sensor array composed of Gr/PDMS composite sensors (show in Fig. 2a). Compared with the classic platinum (Pt) thin-film temperature sensor (TCR = ${ }0.0055\,{\text{K}}^{ - 1}$) in the same measurement of the resistivity under the change of the ambient temperature (30–110 °C), when the graphite volume fraction is 15% and 25%, the TCR of the composite materials is $0.286\,{\text{K}}^{ - 1}$ and $0.042\,{\text{K}}^{ - 1}$, the results prove that the sensitivity of the Gr-PDMS composite material is higher. Huang and others have researched and fabricated a graphite-filled polyethylene oxide (PEO) and polyvinylidene fluoride (PVDF) composite material [68], (Fig. 2b) which can be easily attached to the surface of the human body with variable curvature through a simple spin-coating process. A temperature sensor with a sensing range of 25.0–42.0 °C, with a high resolution of 0.1 °C and high cycle stability, excellent anti-interference ability (including anti-bending and waterproof), and can maintain the sensor within 1 month. It can measure the temperature of the armpits continuously for a long time. Its excellent mechanical properties maybe because the Gr particles as fillers in the composite move less under different curvatures. The thermal performance is related to the thermal expansion of the polymer. As a one-dimensional nanomaterial, carbon nanotubes connected to form a spatial topological structure [69]. The diameter is generally 2–20 nm. It has many abnormal mechanical properties (elastic modulus up to $1 \,{\text{TPa}}$), electrical properties, including electrical conductivity up to $10^{4 } \;{\text{S}}\,{\text{cm}}^{ - 1}$ [70], intrinsic carrier mobility (${10,000}\, {\text{cm}}^{2} \,{\text{V}}^{ - 1} \,{\text{S}}^{ - 1}$) [71]. In recent years, with the deepening of research on carbon nanotubes and nanomaterials, their broad application prospects have also been continuously revealed. Carbon nanotubes have excellent heat transfer performance, and CNTs have a considerable aspect ratio, so they are along the length direction. The heat exchange performance is very high, and the application of flexible temperature sensors is also constantly innovating, including excellent design ideas and performance [72]. The composites formed by carbon nanotubes and poly(3,4-ethylene dioxythiophene)-poly(styrene sulfonate)(PEDOT: PSS) were studied, and it found that the performance of multi-walled carbon nanotubes (MWCNTs) and single-walled carbon nanotubes (SWCNTs) were not the same [73]. At the same temperature, the composite materials formed by MWCNTs and PEDOT: PSS later, while reducing the impedance of the composite, the composite also reduces its sensitivity to temperature and humidity [74]. Kim et al. [75] used a wet-spinning process and also used a PEDOT: PSS composite. The SWCNTs composited with it can significantly enhance the composite's electrical conductivity and power factor and improve the composite performance (Fig. 2d).

Conventional methods of making biological materials rely on reverse engineering of biological structures, bionics, and biological inspiration. Biological structures often outperform human-made materials. For example, higher plants sense temperature changes with high responsiveness. Shown in Fig. 2c, Giacomo et al. [76] proposed a biomimetic material with excellent temperature response sensitivity made of isolated plant cells and carbon nanotubes (MWCNT) using plant nano-biomimetic technology. Using CNTs as a channel to connect cells directly, creating a bionic material with an effective temperature resistivity (TCR) of $- \,1730\% \,{\text{K}}^{ - 1}$, which is two orders of magnitude higher than the current best sensor, and the monitoring temperature range is 35–75 °C. Many types of research on temperature sensors can achieve high sensitivity in a narrow temperature range, but in a wide temperature range (40 K), the response performance is insufficient. Also refer to the biological temperature sensing structure of snakes in nature- snakes' pit membrane, the biological membrane has exceptionally high temperature and distance sensitivity. It can use to locate warm-blooded prey at a certain distance. They used the addition of Ca^2+^ ions to the pectin film to mimic the snake's cheek film's sensing mechanism to prepare a pectin hydrogel temperature-sensitive material added with CaCl_2_ [10]. It is possible to map and monitor temperature sources with a sensitivity of < 10 mK and sense warm objects at a certain distance. Combining plant cells or imitating animal biological structures provides new ideas and makes the research direction of flexible sensors more extensive.

Graphene is a two-dimensional hybrid carbon layer with a hexagonal honeycomb lattice [77, 78]. There are a large number of delocalized electrons, making its unique charge transport allowing high carrier concentration (${10}^{{{33}}} \,{\text{cm}}^{ - 2}$), under certain environmental conditions, the mobility exceeds ${10}^{5} \,{\text{cm}}^{2} \,{\text{V}}^{{ - {1}}} \,{\text{s}}^{ - 1}$. And the spatial structure can provide abundant bits dots. Functional groups are modified to meet various application requirements. Simultaneously, it has high fluidity, excellent thermal conductivity, excellent transparency, mechanical properties up to $1 \,{\text{TPa}}$ elastic modulus, chemical stability, and biocompatibility [79]. It has attracted much attention in the application fields of various electronic devices [80]. Transparent graphene oxide (GO) or reduced graphene oxide (rGO) with excellent electronic and mechanical properties is a product with a layered structure formed by graphite powder after oxidation or further reduction of GO [81, 82]. The surface contains hydroxyl groups, carboxyl groups, and rings. Many functional groups, such as oxy groups, are easily modified and sensitive to environmental conditions, including humidity, temperature, and chemical substances, and visible response characteristics [54, 83]. However, the low conductivity of graphene oxide (GO) is not suitable for electronic devices. The reduced graphene oxide (rGO) is synthesized by thermal reduction to improve conductivity. Its excellent temperature sensitivity is also required for flexible temperature sensors [54, 84, 85]. Graphene nanowalls (GNWs) are grown into graphene nanosheets perpendicular to the substrate by employing plasma-enhanced chemical vapor deposition (PECVD) technique [77] and polymer-assisted transfer method. The growth process forms a staggered structure to make it have higher strain performance [61, 83]. Excellent mechanical properties have also applied to temperature sensors in research. The use of multi-purpose graphene and its derivatives in electronic skin applications paves the way for flexible temperature sensors with excellent performance, transparency, rich functions, and a simple fabricating process.

Yan et al. [58] fabricated a flexible temperature sensor that uses three-dimensional pleated graphene as the active material and can monitor the temperature in the range of 30–100 °C through a lithographic filtering method [34]. Due to the particularity of the spring-like structure, the temperature change sensing characteristics can characterize at up to 50% strain. The TCR ($- \,1.05 \pm 0.28\% \,{\text{K}}^{ - 1}$) of the sensor under unstrained conditions is higher than the reported silicon nanowire temperature sensor ($0.15 - 0.37\% \,{\text{K}}^{ - 1}$) times. It is worth noting that the thermal index of the thermal material of this structure can be adjusted. The temperature response and recovery can complete within tens of seconds, which increases its applicability compared to traditional rigid ceramic thermistors. Liu et al. [87] used rGO material as the temperature-sensitive material to fabricate a lightweight and low-cost, flexible temperature sensor through printing technology. The monitoring temperature is 20–110 °C, the sensitivity is $0.6345\% \,^{ \circ } {\text{C}}^{ - 1}$, and the response time can reach 1.2 s. With specific stress and strain characteristics, can be attached to a specific curvature surface. Under the same experimental conditions, after comparing reduced graphene oxide (rGO), single-walled carbon nanotubes (SWCNTs) and multi-walled carbon nanotubes (MWCNTs), in the comparison of linearity, sensitivity, mechanical properties, and repeatability, it is found that the performance of the temperature sensor using rGO as the active material is the most balanced, providing ideas for the large-scale preparation of electronic skin in the future. Sadasivuni et al. [54] proposed a composite film using cellulose as the matrix and thermally rGO as the filler to produce a flexible and efficient monitoring temperature range of 25–80 °C, depending on temperature changes.(show in Fig. 2e) Capacitive flexible temperature sensor with a linear relationship. Compared with the standard commercial platinum temperature sensor, the temperature sensor will not cause pollution due to metal corrosion phenomena over time and can maintain stability for a long time. Trung et al. [17] fabricated a transparent and stretchable (TS) flexible temperature sensor through a simple spin coating method and lamination technology. The temperature sensing layer formed by rGO nanosheets and an elastic polyurethane (PU) substrate. Composite material formation. The novelty of the electronic device is that each layer of material in the structure is TS and can be easily directly coated on a transparent and stretchable substrate. It can then be easily attached to the human body. The device can detect temperature changes as small as 0.2 °C. After being stretched 10,000 times with a 30% strain, it has almost no effect on the temperature response. The device can still be used when the strain is 70%. Yang et al. [86] proposed using plasma-enhanced chemical vapor deposition (PECVD) technology to grow a special interlaced 3D conductive GNWs network structure on copper foil (Fig. 2f), combined with a polymer-assisted transfer method and PDMS to form an ultra-sensitive wearable temperature sensor. The thermal response far exceeds that of traditional temperature sensors. The excellent ductility and thermal sensitivity of GNWs combined with the large expansion coefficient of PDMS enable the sensor to monitor the temperature change of 25–120 °C with an accuracy of 0.1 °C, a response time 1.6 s and a recovery time of 8.52 s, and maintain stability within months. TCR reaches $0.214\,^{ \circ } {\text{C}}^{ - 1}$, which is two orders of magnitude higher than the standard commercial platinum temperature sensor ($39.2 \times 10^{ - 4 } \,^{ \circ } {\text{C}}^{ - 1}$). The use of carbon materials in flexible temperature sensors has promoted its application potential in health monitoring, wearable devices, robotics, human–machine interfaces, and artificial skin.

##### Metal and Metal Oxide

**Fig. 3:**
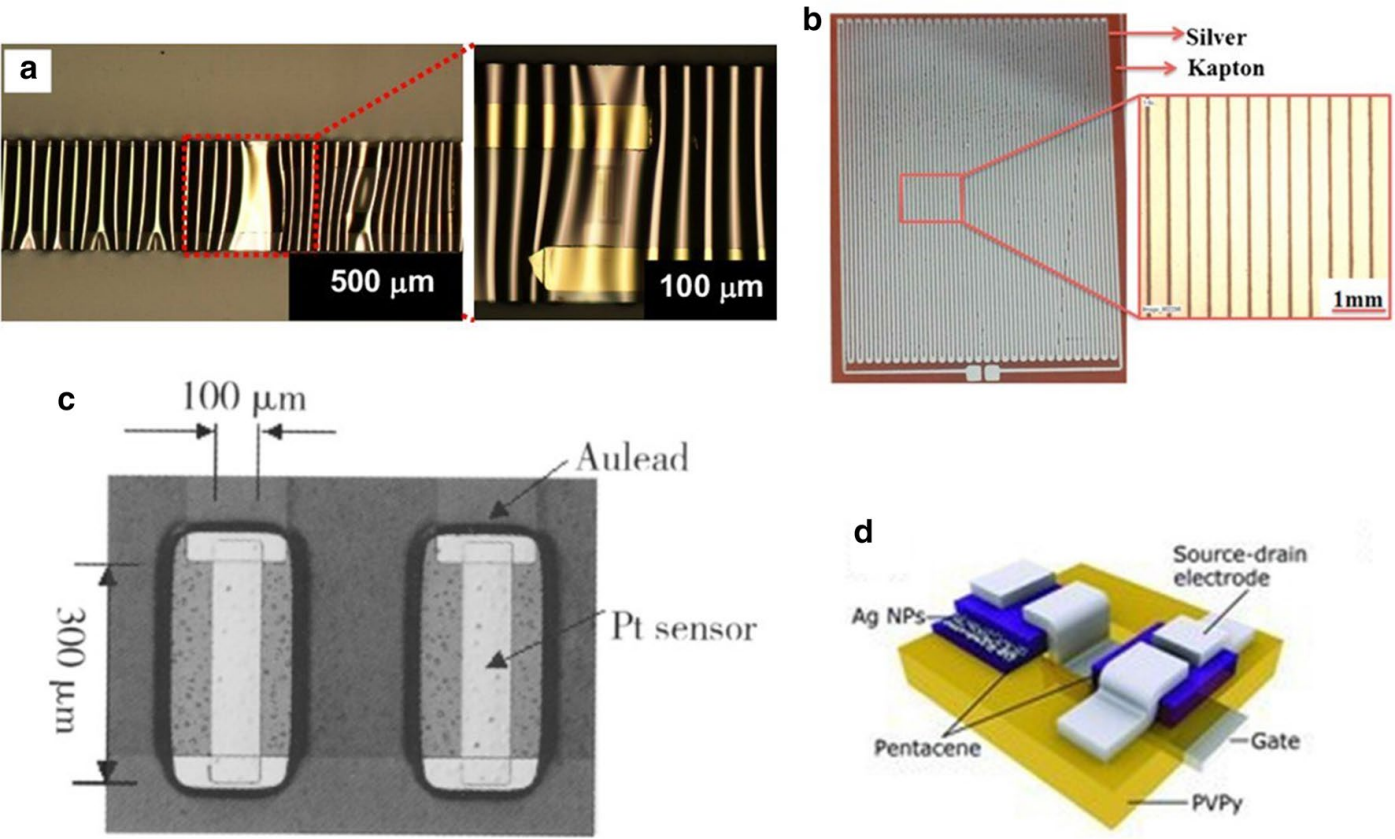
Various flexible temperature sensors based on metal materials. **a** Stretchable sensors on top of a PDMS substrate with periodically buckled patterns [99]. **b** Photographs of inkjet printed silver temperature sensor on Kapton substrate [102]. **c** An image of the temperature sensors [6]. **d** The schematic diagrams of the (one transistor)–(one thermistor) temperature sensor [101]

Metal materials are generally conductive materials such as gold(Au) [88–90], silver(Ag) [91, 92], copper(Cu) [93],and platinum(Pt) [55, 94] (in Fig. 3c), nickel (Ni) [95], and aluminum (Al) [96], which mainly used as electrodes and wires of sensors. Compared with the traditional rigid metal temperature sensor, the flexible metal temperature sensor has high mechanical flexibility, can be easily attached to highly curved surfaces, and is more suitable for detecting small temperature changes and distributions in a small range happening. As far as the current printing process is concerned, some metal materials are sensitive to temperature and have good conductivity, and are made of conductive metal nano-ink [97], nano-filler [95], nano-wire [98], and patterned film to make an active temperature-sensitive layer [19], which is widely used in flexible temperature sensor.

The most common sensitive materials in typical temperature sensors are Pt and Au. Bin et al. [6] used the Micro-Electro-Mechanical System (MEMS) technology and proposed platinum as the sensing material deposited on the PI film after a peeling process. After lamination forming the required pattern, it is used to monitor the temperature change of 20–120 °C, and the TCR value is $0.0032\,^{ \circ } {\text{C}}^{ - 1}$. Compared with the expensive platinum, Au has better conductivity and flexibility. To realize the device's performance can’t be affected by tensile strain, Yu et al. [99] cleverly used sputter deposition on a pre-stretched PDMS flexible substrate Chromium(Cr)/Au thin film (in Fig. 3a), and a reversible bendable and stretchable flexible temperature sensor is fabricated through a photolithography process. When the device's maximum stretch is 30%, the device's performance will not change. The research improves the defect of poor tensile resistance of flexible sensors. Dankoco et al. [100] used an organic silver composite ink to deposit silver lines on a polyimide film smoothly and evenly by inkjet printing (see in Fig. 3b). It made a measurable body surface temperature of 20–60 °C can be a bent and flexible temperature sensor. The average sensitivity is $2.23 \times 10^{3} \,^{ \circ } {\text{C}}^{ - 1}$. However, the sensor has < 5% hysteresis. Ren et al. [101] proposed a flexible temperature sensor with high thermal resolution (dynamic range = 10 bits) based on the integration of silver nanoparticles (NPs)/pentacene thermistor and organic thin-film transistor (OTFT) with a temperature range of 15–70 °C. The research tested the high dependence of composite materials on temperature and proved the feasibility of silver nanoparticles (NPs) in thermistor applications. High dynamic range sensors are also suitable for large-area sensor arrays and electronic skin. Jeon et al. [95] developed a flexible temperature sensor designed with a mixture of semi-crystalline polyethylene (PE) and polyethylene oxide (PEO) polymers filled with nickel particles. Among them, when the concentration of nickel particles exceeds the permeation threshold of nickel fillers, high conductivity ($40 \,{\text{S}}\,{\text{cm}}^{ - 1}$) can be obtained. With the large positive temperature coefficient (PTC) unique to the binary polymer mixture, the fabricated sensor can not only provide an adjustable sensitive temperature range while maintaining the stability of the thermal cycle to achieve a repeatable temperature response process. The sensor has three orders of magnitude higher sensitivity ($0.3\, {\text{V}}\,^{ \circ } {\text{C}}^{ - 1}$) than standard thermocouples and wireless transmission function, but there is a significant error of ± 3.1 °C. It is worth noting that they want to combine wireless sensing technology with printable materials to achieve a wide range of applications.

Metal oxide is also important active materials and have used widely in temperature sensors. The high temperature coefficient of resistance of metal oxide materials can improve temperature sensing performance. The thermal sensitivity of metal oxide semiconductors is a phenomenon in which the semiconductor compound changes at different temperatures and the resistance value changes. Liao et al. [103] reported a high-sensitivity temperature/mechanical dual-parameter sensor containing inorganic thermal material vanadium dioxide (VO_2_), which based on PET/vanadium dioxide fabricated by transfer printing technology (VO_2_)/PDMS multilayer structure. The VO_2_ layer deposited by polymer-assisted deposition (PAD) technology is etched and attached to the PDMS film. It can detect the temperature in the range of 270–320 K. The temperature sensing performance with a resolution of 0.1 K attributed to the high TCR of the VO_2_ material. In order to accurately map body surface temperature changes, Huang et al. [91] developed an inkjet printing process. Using nickel oxide (NiO) to generate a stable nanoparticle ink on the PI film. The tiny square NiO film is printed at the ends between the silver conductive tracks to fabricate temperature sensors quickly. It can still maintain performance under the bending test and has a similar response speed as a thermocouple. The extensive research on metal and metal oxide has laid the foundation for the application of metal materials in the field of flexible temperature sensor based on the thermal properties of metal materials and shown interesting exploration.

##### Polymers and Organic Materials

Polymers are the most used materials in flexible sensors. In addition to being used as substrates or active agents, thermally sensitive composite materials with mechanical flexibility, lightweight, transparency, stable performance, easy processing, and low fabricating costs are flexible. The application in the temperature sensor has attracted much attention. Thermosensitive polymers often used in temperature sensors include poly(3,4-ethylene dioxythiophene)-poly(styrene sulfonate) (PEDOT: PSS) [16, 104–106], poly(3-hexyl thiophene) (P3HT) [107], polypyrrole (PPy) [57], pentacene [101], poly (N-isopropyl acrylamide) (pNIPAM) [108], poly(vinylidene fluoride) (PVDF) [109–113], etc.

**Fig. 4:**
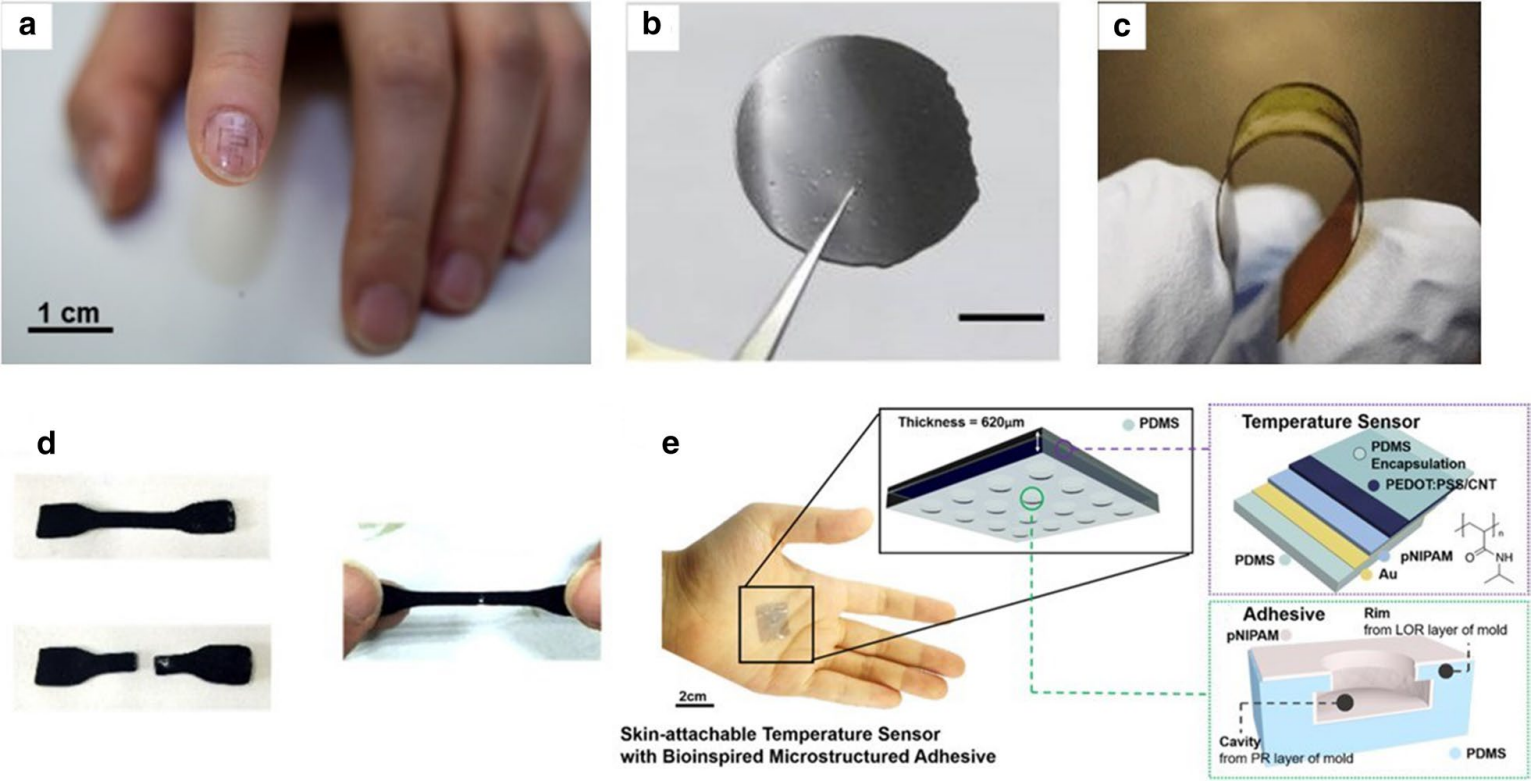
Various flexible temperature sensors based on thermosensitive polymer. **a** A sample with two temperature sensors on a fingernail [115]. **b** Photograph of a film of copolymer with graphite filler (scale bar, 1 cm) [116]. **c** Photograph of a Te-nanowire/P3HT-polymer composite device on a flexible Kapton substrate [107]. **d** Photos of the DN hydrogels self-healing process [121]. **e** Photographic image of the fabricated temperature sensor attached onto palm skin and the overall schematic illustration of the octopus-mimicking microstructured adhesive [108]

Based on a circuit design strategy [114] that can improve the accuracy and robustness of a stretchable carbon nanotube temperature sensor, Zhu et al. [115] used differential readout technology to compare the composition of the active, sensitive layer of a stretchable temperature sensor based on OTFTs (see Fig. 4a). Among them, Polystyrene- block-poly(ethylene-ran-butylene)-block-polystyrene (SEBS) with azide-crosslinke and Poly(diketopyrrolopyrrole-[3,2-b]thieno[2′,3′:4,5]thieno[2,3-d]thiophene]) (PDPPFT4) and Poly(isoindigo-bithiophene) (PII2T) these two organic semiconductors (OSCs) are blended and spin-coated on the gate dielectric, CNTs are used as electrodes, and the temperature measurement range is 25–55 °C. Inside, the temperature coefficients of the two sensors are $- \,2.89\% \,^{ \circ } {\text{C}}^{ - 1}$ and $- \,4.23\% \,^{ \circ } {\text{C}}^{ - 1}$, respectively. When the uniaxial strain range is 0–30%, the errors are < 1 °C and < 1.5 °C, respectively, further show the feasibility and generalizability of differential readout method and OSCs in stretchable sensors are discussed. Yokota et al. [116] reported a large-area super-flexible temperature sensor based on a semi-crystalline acrylate polymer/graphite composite material that can be measured at multiple points and can be printed (in Fig. 4b). Between 25 °C and 50 °C, it shows noticeable resistance changes at this temperature, which is suitable for measuring the physiological temperature changes of the human body. It has stable thermal cycle stability, the sensitivity of up to 20 mK, and a high-speed response time of less than 100 ms. In in vivo experiments, the stable changes in the rat's lungs' core temperature measured, but the high resolution of the sensor proved to be 0.1 °C. The sensor array based on the above characteristics realizes the dynamic visual, thermal imaging demonstration of the spatial temperature change. However, the air permeability of the equipment components is not right, and the long-term wear is one of the problems to be solved.

Self-supplied energy has always been the focus of many people's attention [117]. The realization of self-supplied energy by flexible equipment will significantly reduce the equipment's need for external energy, making flexible equipment more portable and more straightforward [118]. Among them, the thermoelectric polymer materials are the realization of self-supplied energy required. Yang et al. [107]developed a flexible thermoelectric nanogenerator (TENG) based on a clean composite thermoelectric material formed by Te-nanowires grown at room temperature and poly(3-hexyl thiophene) (P3HT) polymer (shown in Fig. 4c). TENG can generate electricity only with a temperature difference of 55 K. Because of the characteristics of thermoelectric materials, TENG can be used as a flexible temperature sensor to monitor the temperature difference of the entire device, and use human body temperature as an energy source to directly power the sensor. The monitoring sensitivity at room temperature is 0.15 K. Besides, they also demonstrated another self-powered temperature sensor with a response time of 0.9 s and a minimum temperature change of 0.4 K at room temperature. The small temperature resolution makes the sensor device can monitor the temperature change of the fingertip. Self-supplied can make flexible sensor equipment more independent and reduce weight. It is also a possibility for the development of flexible sensors in the future (Table 2).

**Table 2:** Flexible temperature sensors application and key parameters

Materials	Fabrication methods	Performance						Application
		Sensitivity	Measurement range (°C)	Response time	Accuracy/resolution (°C)	Linearity	Repeatabilityand durability	
Gr/PDMS [67]	Dispensing technology	$0.286 \,{\text{K}}^{ - 1}$	30–110	–	-	Yes	–	Robotic applications
Gr/PEO/PVDF [68]	Coating techniques	–	25–42	26 s	0.1	–	2000 cycles 1 month	Medical diagnosis; temperature monitoring
MWCNT/BY-2 cells [76]	Plant nanobionics	$- \,1730\% \,{\text{K}}^{ - 1}$	35–75	–	–	–	–	Thermal and distance sensors
Graphene/PDMS [58]	Lithographic filtration methods	$- \,1.05\% \,{\text{K}}^{ - 1}$	30–100	–	–	Yes	100 cycles	Wearable temperature sensing applications
rGO/PET [87]	Printing technology	$0.635\% \,^{ \circ } {\text{C}}^{ - 1}$	30–100	1.2 s	–	Yes	3 cycles	Robot skins; IoT
rGO/Cellulose [54]	Cast technology	–	25–80	–	–	Yes	–	Wearable devices
rGO/PU [17]	Coating techniques	$1.34\% \,^{ \circ } {\text{C}}^{ - 1}$	30–80	–	0.2	Yes	10,000 cycles	Wearable skin electronics
GNWs/PDMS [86]	PECVD & Polymer assisted transfer methods	$0.214\,^{ \circ } {\text{C}}^{ - 1}$	25–120	1.6 s	0.1	Yes	10 cycles 2 months	Human health monitor
Pt/PI [6]	MEMS technology	$0.32\% \,^{ \circ } {\text{C}}^{ - 1}$	20–120	–	–	Yes	3 cycles	Biomedical applications
Au/Cr/PVA [99]	Microlithography	$2.5\% \,^{ \circ } {\text{C}}^{ - 1}$	25–45	3.7 ms	0.008	Yes	100 cycles	Stretchable electronics
Ag/PI [102]	Printing technology	$0.223\% \,^{ \circ } {\text{C}}^{ - 1}$	20–60	–	–	Yes	40 days	Printed medical applications
Ni/PE/PEO [95]	Liqid phase mixing	$0.3\,{\text{V}}\,^{ \circ } {\text{C}}^{ - 1}$	35–42	–	2.7	–	100 cycles	Medical diagnostics
OA/BA/Graphite/PI [116]	Printing technology	20 mK	25–50	< 100 ms	0.1	–	100 cycles	Health diagnostics; wearable devices
TE-nanowires/P3HT [107]	Drop casting	0.15 K	–	17 s	0.4	Yes	–	TENG
pNIPAM/PEDOT:PSS/CNTs/PDMS [108]	Coating techniques & Lithography	$2.6\% \,^{ \circ } {\text{C}}^{ - 1}$	25–40	139 s	0.5	Yes	500 cycles 12 h	Disease diagnosis; e-skin
rGO/P(VDF-TrFE) [113]	Coating techniques	–	30–80	–	0.1	Yes	1000 cycles	Electronic skin; human–machine interface
PEDOT:PSS/ PU [23]	Dipping method	0.1 K	25–75	< 2 s	0.1	Yes	10,000 cycles	Robotics and health-monitoring products
CaCl_2_/pectin [178]	Liqid phase mixing	10 mK	8–39	–	–	Yes	–	Biomedical applications
VO_2_/PDMS [103]	Printing technology	$- \,1.12\% \,{\text{K}}^{ - 1}$	3.15–46.85	< 2 s	0.1	Yes	10,000 cycles	Wearable AI elements
MWCNT/PVDF yarn [111]	Dip-coating techniques	$0.13\% \,{\text{K}}^{ - 1}$	30–45	–	–	Yes	200,000 cycles	Smart textiles
GNRs/paper [171]	Pen-on-paper	$1.27\% \,{\text{K}}^{ - 1}$	30–80	0.5	0.2	Yes	5000 cycles	Disposable sensors
PEDOT:PSS/ paper [177]	Dip-coating techniques	$658.5\, \Omega \,^{ \circ } {\text{C}}^{ - 1}$	30–42	–	–	Yes	–	Medical diagnostics
PEDOT:PSS/CNTs/PET [170]	Printing technology	$0.85\% \,^{ \circ } {\text{C}}^{ - 1}$	30–55	< 50 ms	–	Yes	92 days	Health monitoring

Low-cost, environmentally friendly, easy-to-obtain, and process materials with excellent biocompatibility have always been an essential condition for human beings to pursue to meet mass production continuously [119]. There is such an almost inexhaustible biological material–cellulose, which has excellent properties. Its elasticity and other advantages also play an essential role in flexible sensor devices and can be used as a flexible substrate. Polypyrrole (PPy) is a linear biocompatible polymer with excellent electrochemical stability and rapid response. Mahadeva et al. [57] reported a method based on in-situ polymerization-induced adsorption that combines unique cellulose and nano-thickness polypyrrole (PPy) excellent electrical properties to form a temperature and humidity sensitive composite. The material used to fabricate an environmentally friendly, low-cost, bio-adaptable flexible temperature sensor. Because of the sensitivity of materials to humidity, as the temperature increases, the sensor's capacitance also increases. Hydrogels have received continuous attention in recent years of research [120, 121]. Because of its good self-healing ability, excellent toughness and stretchability, and biological adaptability, it has aroused great research interest in application fields such as flexible electronics, health monitoring, and biomedical diagnosis [122, 123]. However, ionic hydrogel, as a good ion conductor, can respond to a variety of stimuli, hydrogels with weak mechanical strength, and reduced temperature sensitivity present challenges in applying flexible temperature sensors. To solve the disadvantages of traditional hydrogels, An et al. [121] proposed a double-mesh ion-conducting double-network (DN) hydrogel with excellent temperature sensors' self-healing properties. The DN hydrogels self-healing process is shown in Fig. 4d. The addition of carbon nanotubes with high thermal conductivity to the hydrogel with dynamic physical crosslinking and high conductivity hydrophobic association network and ion association network improves the temperature sensitivity of DN hydrogel. The linear hydrogel temperature sensor can perfectly fit the surface of complex objects and produce sensitive resistance changes. The research and development of this material expand hydrogels' application in the fields of biomedicine and flexible electronics.

In recent research, PEDOT: PSS is a new type of organic conductive polymer that often used in printable, flexible temperature sensors [124]. Generally speaking, PEDOT: PSS has the advantages of high conductivity ($10^{3} \,{\text{S}}\,{\text{cm}}^{ - 1}$), excellent thermoelectric performance [125–128], strong stability [123, 129], and transparency when doped [60]. Most polymers are p-type semiconductors. By adding some solvents, such as dimethyl sulfoxide(DMSO) [130] or polyhydroxy organic compounds, such as ethylene glycol [131], the conductivity rate of the polymer can be increased dozens of times or even hundreds of times. Harada and colleagues used different composite ratios of CNTs and conductive PEDOT: PSS solutions to produce a series of composite heat-sensitive films with temperature sensitivity within $0.25 - 0.78\% \,^{ \circ } {\text{C}}^{ - 1}$ through various printing processes [11, 132–134]. The sensing performance is better than the typical metal temperature sensor. Some of the devices exhibited a near body temperature resolution of less than 0.1 °C or fast response time of 90 ms. The research team used various printing methods to create a variety of flexible temperature sensors with different structures and outstanding performance, in addition to enriching the flexible temperature sensors applications in medical and health, wearable devices. Have also triggered everyone thinking about printable, flexible sensors. The fabricating details of printable, flexible temperature sensors will be expanded in the next unit. In addition to changing the composite ratio will affect the performance of the composite film, the structural improvement will also optimize the properties of the same composite film. As Fig. 4e shown, Oh et al. [108]demonstrated a biological material made by a photolithographic stripping process and a spin coating process, inspiringly imitating the adhesion structure of octopus foot sucker. It is a high-sensitivity resistor temperature sensor composed of poly(n-isopropyl acrylamide) (pNIPAM) temperature-sensitive hydrogel, PEDOT: PSS and CNTs. The device has a sensitivity of $2.6\% \,^{ \circ } {\text{C}}^{ - 1}$ in the temperature range of 25–40 °C, and can accurately detect skin temperature changes of 0.5 °C. Because of the microstructure similar to the suction cup and its viscosity, the device has a certain degree of resistance to bending, non-irritating, long-lasting, and reusable binding effect.

**Fig. 5:**
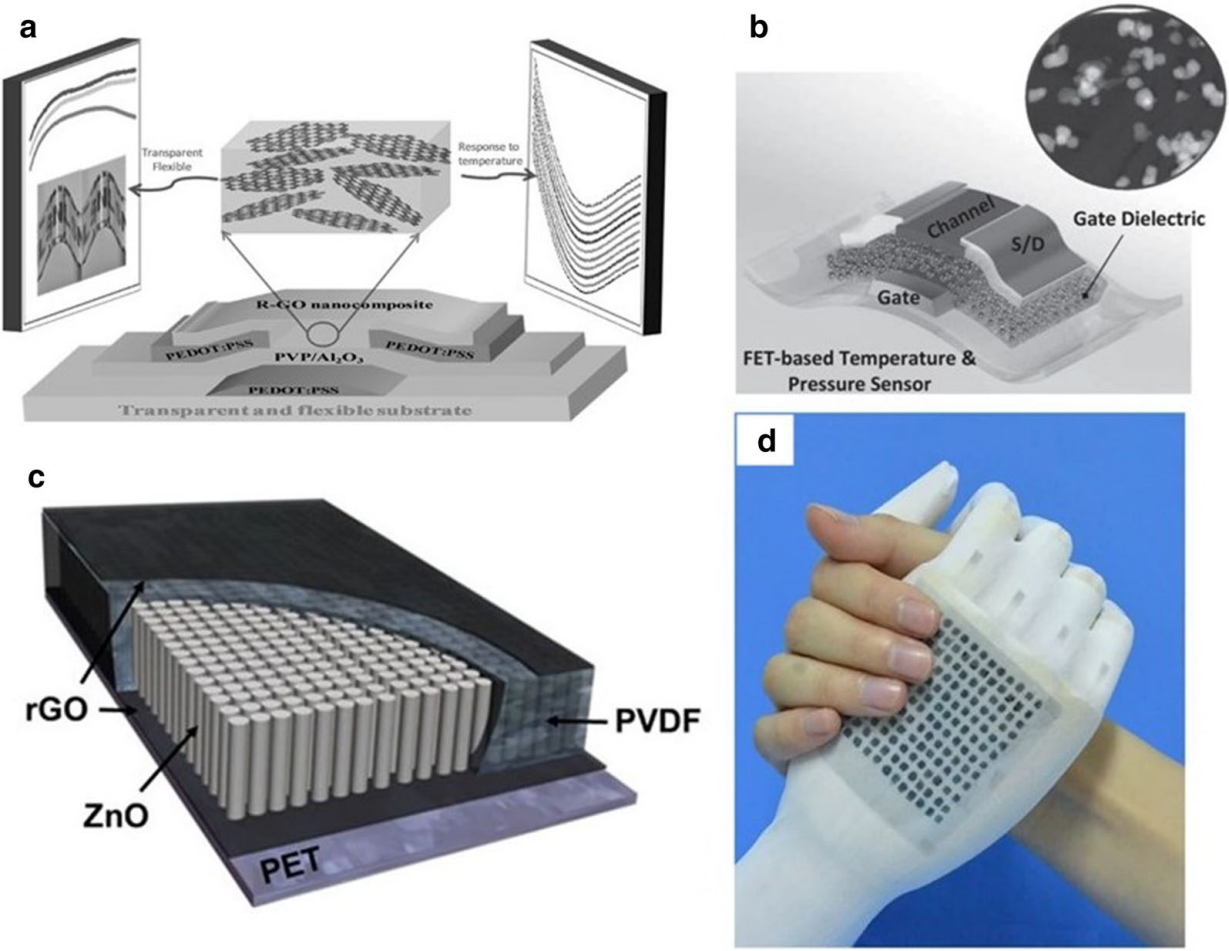
Flexible temperature sensor containing PVDF material. **a** Schematic of transparent, flexible rGO/P(VDF-TrFE) nanocomposite FET. The schematic illustrates structural, optical (transparent) and electrical (response to temperature) properties of the transparent, flexible R-GO/P(VDF-TrFE) nanocomposite FET [113]. **b** The structure of physically responsive field-effect transistor (physi-FET) with the bottom-gated and top-contact structure, where the gate dielectric is comprised of nanocomposite of P(VDF-TrFE) and BaTiO_3_ nanoparticles and the channel is organic semiconductor of pentacene [112]. **c** Schematic diagram of the ZnO/PVDF composite film and rGO electrodes [110]. **d** Photograph of the flexible MFSOTE matrixes [23]

Transparent and scalable nanocomposite field-effect transistor based on polyvinylidene fluoride (PVDF) and its copolymer polyvinylidene fluoride (P (VDF-TrFE)) with high stability, strong mechanical properties, and low distortion, it is often used in pressure sensing, strain sensing, and infrared (IR) light-sensing devices [135, 136]. Interestingly, researchers have found that this type of device is also highly responsive to infrared radiation from the human body, so it is predicted to be used to monitor the human body's physiological temperature changes [137]. Trung and colleagues, based on previous research experience [113, 138], use rGO/(P(VDF-TrFE)) composite sensing active layer as the channel through a simple spin coating process, integrated PEDOT: PSS can fabricate adjustable flexible field-effect transistor (FET) temperature sensor [139]. The film's temperature response and transparency can adjust by changing the concentration of rGO and the thickness of the composite film (Fig. 5a). The sensor can monitor temperature changes from 30 to 80 °C. With 0.1 °C resolution and monitoring ability, and super high-temperature response, excellent temperature sensing performance verifies the feasibility of the application of pyroelectric polymer materials in the field of softcore temperature sensors. Similarly, Tien et al. [112] in order to realize that the sensor can collect pressure and temperature signals without mutual interference, they proposed the use of field-effect transistor (FET) sensing platform to change the material of the response sensitive layer, based on their previous the research concluded that a mixture of polyvinylidene fluoride (P(VDF-TrFE)) and BaTiO_3_(BT) nanoparticles (NPs) is used as piezoelectric (see Fig. 5b), pyroelectric gate dielectric and pentacene are used as organic semiconductor channel for pressure thermal resistance directly integrated into the FET platform when the flexible sensor is under multiple stimuli, it decouples the output signal and minimizes the signal interference of strain coupling. The FET sensor can disproportionately present strain and temperature at the same time. The FET sensor array can also visually respond to stimuli, exhibiting the advantages of low energy consumption and low failure, which shows a possibility for the application of large-area multi-modal flexible sensors in the field of electronic skin in the future. Flexible multi-parameter OFET devices that can be printed and fabricated in a large area have excellent application potential in biomedical monitoring, infrared imaging, and electronic skin.

In addition to the decoupling method to reduce or eliminate signal interference, in order to solve the problem of mutual interference of multi-parameter flexible sensor signals, Lee et al. [110] proposed a method of inferring temperature based on the recovery time of the resistance change signal, so that the semiconductor zinc oxide (ZnO) nanostructure is mixed into the substrate polyvinylidene fluoride (PVDF) as a filler (As shown in Fig. 5c) to make a highly sensitive multifunctional sensitive layer that can collect temperature and pressure signals at the same time. Among them, the semiconductor ZnO can increase the dielectric constant of PVDF, and it also has thermal stability. Zhang et al. [23] reported a dual-parameter flexible sensor based on a self-powered microstructure-frame-supported organic thermoelectric (MFSOTE) material. The Fig. 5e show the flexible MFSOTE matrixes. By converting the signal changes caused by temperature and pressure stimulation into two independent electrical signals, the temperature and pressure simultaneously sensed. This unique material shows excellent temperature sensing characteristics. The monitoring temperature range is 25–75 °C, The resolution can achieve < 0.1 K, the response time under 1 K temperature difference is < 2 s, and it can also be adjusted according to different substrates to meet the sensing needs.

### Fabrication

With the increasing requirements for flexibility, multi-function, simple fabricating, and high sensitivity of electronic devices, the exploration and discovery of flexible sensor fabricating methods with a lightweight, simple process, low cost, and large-area fabricating have always been what researchers are keen [140]. This section mainly summarizes the recently reported and feasible fabricating strategies of flexible temperature sensing elements and discusses the key processes to improve their performance.

#### Thin Film Deposition

The thin film preparation method can be divided into vapor deposition and phase deposition according to the phase of the material used. The phase deposition includes spin-coating and inkjet printing processes mentioned later. In contrast, the vapor deposition depends on whether the deposition process contains the chemical reaction process divided into physical vapor deposition (PVD) and chemical vapor deposition (CVD).

**Fig. 6:**
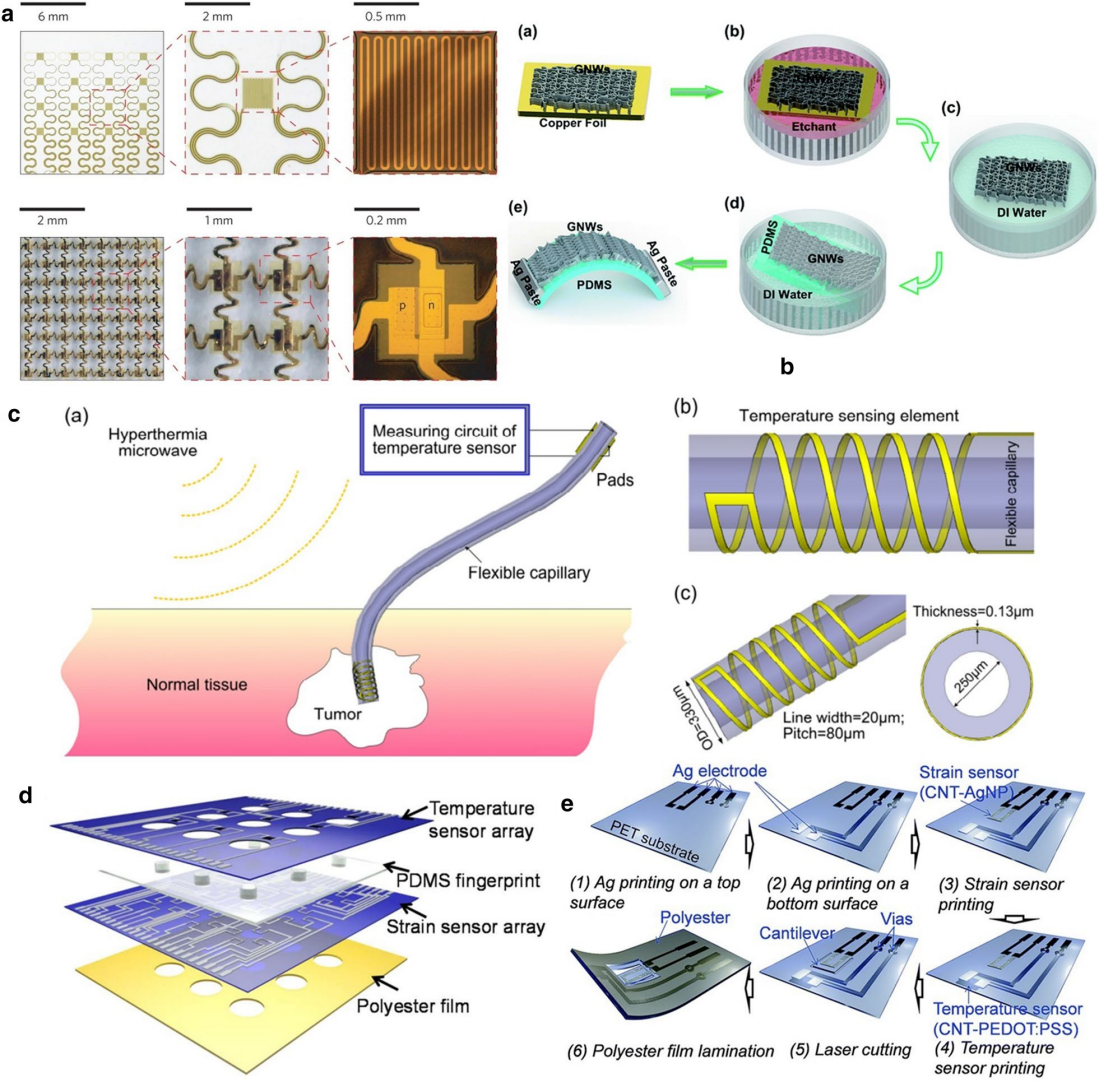
Fabrication method of flexible temperature sensor method of flexible temperature sensor. **a** Top: Optical images of a 4 × 4 TCR sensor array integrated on a thin elastomeric substrate with magnified views of a single sensor. Bottom: Optical images of a 8 × 8 Si nanomembrane diode sensor array integrated on a thin elastomeric substrate with magnified views of a single sensor [145]. **b** Schematic process for the fabrication of GNWs/PDMS temperature sensors [86]. **c** Sketch of the implantable micro temperature sensor on polymer capillary and its application. The head spiral sensing element is fabricated by photolithography [157]. **d** Schematic for each layer of the e-skin device [132]. **e** Schematic of the e-skin fabrication process on a PET substrate using a printing method [134]

PVD is to depositions or atoms generated by physical methods on a substrate under vacuum conditions to form a thin film, which generally used to prepare electrodes or active metal layers [141, 142]. Common deposition methods include vacuum evaporation, vacuum sputtering, and ion plating. Among them, the metal target ion sputtering refers to the vacuum container, under the action of high voltage 1500 V, the remaining gas molecules are ionized to form plasma, and the cations bombard the metal target under the acceleration of the electric field, causing the metal atoms to sputter on the surface of the sample to form conductive film [143]. Ahmed et al. [144] introduced a Si-temperature sensor based on a flexible PI substrate. They deposited undoped amorphous silicon as a sensing material between metal electrodes formed by radio frequency magnetron sputtering and packaged them. Finally, the temperature sensing element is embedded in the flexible polyimide film, and the sensing performance is not affected. The maximum TCR at 30 °C is $0.0288\,{\text{K}}^{ - 1}$. Webb et al. [145] introduced two ultra-thin, skin-like sensor fabricating methods that are self-assembled on the skin surface in the form of an array to provide clear and accurate thermal performance monitoring. A structure is composed of a temperature sensor array, the sensitive layer formed by the serpentine trace structure of the Cr/Au layer deposited on the PI film by the metal evaporation deposition method, the microlithography technology, and the wet etching technology, and the reactive ion etching and metal deposition for contacts and interconnections complete the array. Another sensor structure uses multiplexed addressing to form a patterned PIN diode sensor design of doped Si nano-film. The sensitivity layer is defined by metal evaporation, photolithography, chemical vapor deposition, and wet etching steps. The two arrays are shown in Fig. 6a. Aluminum phthalocyanine chloride (AlPcCl) is often used as a material for solar cells and humidity sensors. Under the study development of Chani et al. [146] AlPcCl is used as a thermistor and deposited on an aluminum electrode on a glass substrate using a vacuum thermal evaporator. The authors found that the AlPcCl film has a higher sensitivity to the temperature at 25–80 °C, and annealing can improve sensing performance. In a flexible temperature sensor developed by Bin et al. [6] that uses Pt resistors as the thermosensitive material, Pt is evaporated on the Al layer deposited on the spin-coated PI film, and the Pt layer is patterned as a sensitive the layer is spin-coated and packaged with polyimide material. After hydrochloric acid treatment, a complete flexible temperature sensor is peeled off, which can be used to measure the surface temperature of objects in the biomedical field.

Compared with other thin-film preparation processes, the chemical vapor deposition method can achieve high-purity and high-quality thin films. It can be structured and controlled at the atomic layer or nanometer level [147–149]. The process of synthesizing GNWs film on copper foil by low-pressure radio frequency plasma enhanced chemical vapor deposition (RF-PECVD) technology. Yang et al. [86] developed a flexible temperature sensor based on GNWs/PDMS. The fabricating process is shown in Fig. 6b. They verified GNWs is feasible as an active layer of a temperature sensor, and its thermal response performance exceeds that of a traditional metal temperature sensor. Compared with traditional CVD technology, using PECVD technology [150] under low temperature and low-pressure conditions can effectively improve the deposition rate and film quality. In another study, Zhou et al. used the floating catalyst chemical vapor deposition (FCCVD) method [151] to synthesize the original SWCNT film with a controllable thickness directly. The continuous network of CNTs grown by this method has significant conductivity and a high favorable Seebeck coefficient. After transferring the original SWCNT film to the PET substrate, drop-cast the branched polyetherimide (PEI) ethanol solution, and dry it to obtain an n-type SWCNT film that can be used in the fabricate of flexible thermoelectric modules. Although CVD can achieve the deposition of any material on any substrate, as the demand for simple, low-cost and large-area fabricating nanodevice fabricating technology continues to grow, the fabricating process is complex, high-cost, and toxic CVD growth processes and The time-consuming etching process is being replaced by more suitable flexible electronic device fabricating technology [58].

#### Micro-nano Patterned Fabrication

Thin-film patterning is one of the core technologies of flexible electronics fabricating. It follows the basic idea of removing materials from top to bottom or adding materials from bottom to top in the fabricating industry. Its key technologies are thin-film fabricating, patterning, transfer, replication, fidelity, and other crafts. Flexible electronics require large-area, low-temperature, low-cost patterning technology. Learned from the patterning technology of microelectronics and micro-electromechanical devices. However, at the same time, we must consider the characteristics of flexible electronic devices such as flexible substrates, organic materials, and large areas. The patterning technologies currently available for flexible temperature sensors include lithography, printing, soft etching, nanoimprinting, inkjet printing, laser sintering, transfer printing, nano-direct writing [152], and other processes.

Lithography is a patterning method to realize various and ingenious geometric figures or structures in flexible electronics. The photolithography process involves transferring the pattern on the photomask to the substrate by using the photoresist with different sensitivity and physical and chemical reactions under the light. The photolithography process usually uses photoresist on an insulator (usually a silicon wafer) to pattern the required pattern or structure after spin coating, and further realize it through a stripping process [153, 154]. Because of the photolithography process and the stripping process, high alignment and etching accuracy, simple mask production, and comfortable process conditions can usually achieve high-precision, feature-rich microstructure systems. In the ultra-thin flexible suture application with an integrated temperature sensor and thermal actuator developed by Kim et al. [155], they used photolithography technology to micro-process the equipment, and the fabricated flexible medical equipment has the stable thermal performance. Lithography technology limited by the necessity of available materials and precision equipment. The thickness of processable materials and thin films are limited. It is not suitable for device fabricating processes that require a large number of active materials. Yang et al. [156] proposed a flexible implantable micro temperature sensor, and used surface microlithography to etch the micro flexible temperature sensor on the outer surface of the polymer capillary (The sensing principle diagram of the miniature thermometer is shown in Fig. 6c). Using Pt as a sensitive material has good linearity, and it has a promising future as an implantable temperature sensor in the biomedical field. However, this technology is the foundation of the microelectronics industry and pioneered the era of wearables flexible electronics.

With the development of science and technology, the printing process has expanded from the traditional text and image field to the micro-nano structure patterning field. technology can deposit various materials on various substrates, and the printing process is not harsh on the environment. In a nutshell, printing technology includes letterpress, lithography, gravure, screen printing, and has evolved into soft etching [158, 159], transfer printing, nanoimprinting, and other methods. According to the specific implementation method, the wearable sensor can distinguish the printed part from the non-printed part with the mask help. In mask printing, the pattern to be transferred must be designed in advance and then formed through the mask. The functional, active material can directly be transferred to the substrate or electrode through the functional ink imprinting process [160]. Screen printing is a typical mask printing technique [161]. In the printing process by absolute pressure, the functional ink is transferred to the substrate through a squeegee with a patterned mesh to form a pattern. The unique printing method allows screen printing to achieve fast, large-area low-cost fabricating requirements on flat or curved surfaces. It has been widely used in fabricating sensor working circuits, electrodes, and sensor sensing elements. Compared with photolithography technology, screen printing can produce patterns on various materials. However, its pattern resolution cannot meet complex geometric shapes requirements and is only suitable for making patterns with simple shapes. Yokota et al. [116] stirred and mixed a variety of semi-crystalline polymers with graphite to form a super-flexible temperature-sensitive copolymer for flexible temperature sensors for human physiological temperature monitoring. The super-flexible temperature sensor element is printed by mask printing by sandwiching the copolymer mixed with graphite filler between two interdigital gold electrodes deposited on the PI film and then forming by hot pressing. Yan et al. [58] used a flat-plate suction filter printing method to deposit graphene through a mask, vacuum filter it, and transfer it to the substrate to form a three-dimensional fold pattern structure produce stretchable graphene with a variable thermal index. The thermistor increases the sensing area and stretchability of the sensor. The pre-designed stretchable sensitive material pattern can still maintain sensitive monitoring of temperature when stretched to less than 50%. To achieve the economical fabricating of sensors with a larger area, Harada and colleagues abandoned complex and costly fabricating processes (such as deposition and photolithography). They chose to fabricate a series of multifunctional flexible sensors using only printing processes. The PEDOT: PSS/CNT composite ink printed on the circuit formed by screen printing on the PET substrate through a shadow mask, and there are holes after laser writing (LS) to combine with the lower layer of PDMS. The fingerprint-like structure (see in Fig. 6d) is combined with the screen-printed strain sensor layer to form a flexible sensor array. The deformation and temperature difference caused by the contact contacts achieve a human-like monitoring performance. In another study, also using full printing technology, Kanao et al. [134] proposed a multifunctional flexible sensor array based on a cantilever beam structure (Fig. 6e). They placed strain sensors and temperature sensors on a flexible screen-printed circuit. On both sides of the PET substrate, a patterned shadow mask with a flexible temperature sensor (PEDOT: PSS/CNT composite ink) printed on the screen's electrical contacts printed circuit. The fully printed array sensor used to imitate the sensing characteristics of human skin. When the cantilever beam structure strained, the heat source is closer to the temperature sensor on the substrate's bottom surface to monitor temperature changes more accurately.

**Fig. 7:**
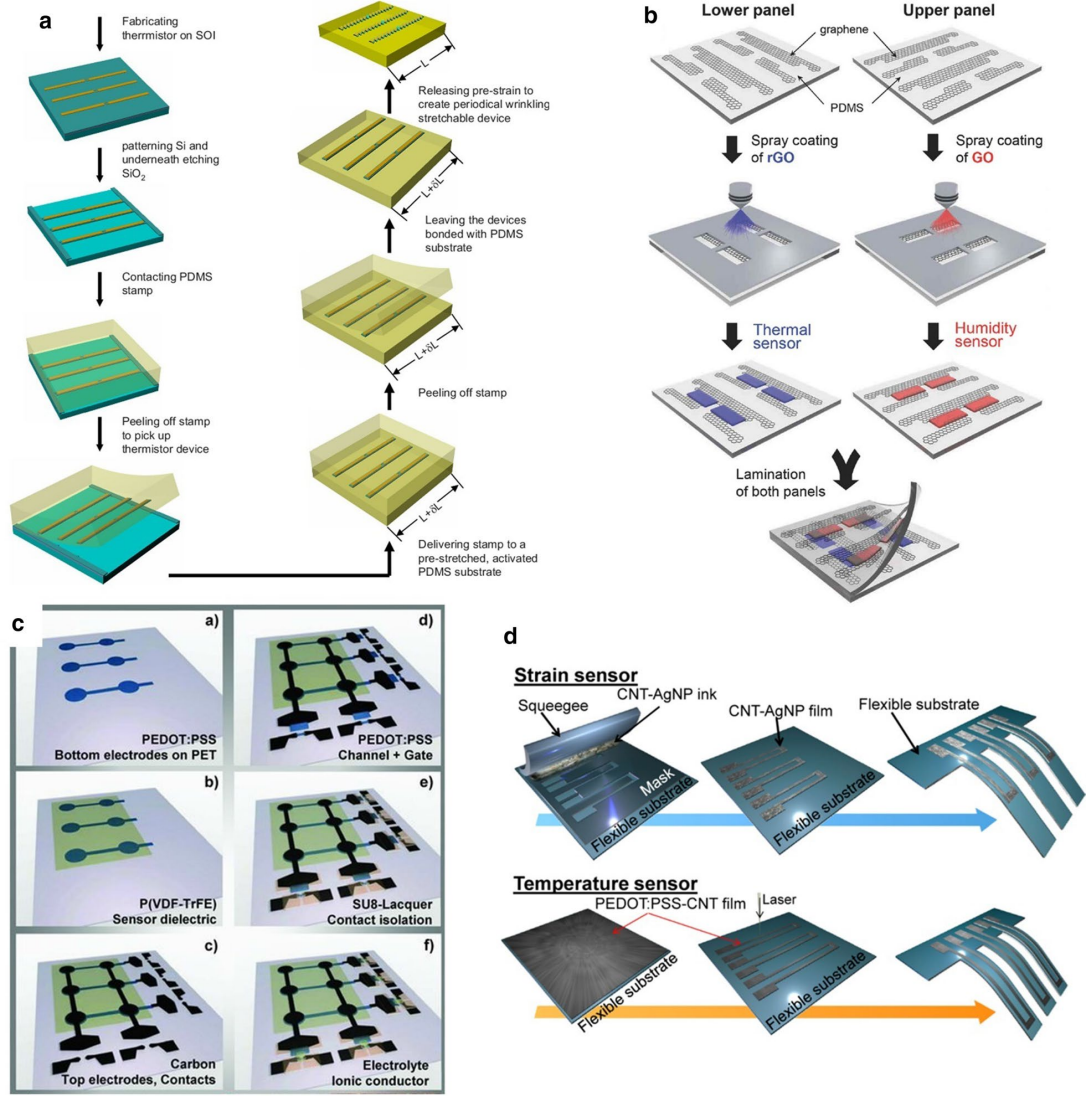
Fabrication method of flexible temperature sensor. **a** Schematic illustration of the fabrication process [99]. **b** Fabrication of the stretchable and multimodal all-graphene E-skin sensor matrix [84]. **c** Process flow illustrating the fabrication of printed ferroelectric active matrix sensor arrays [169]. **d** Multifunctional e-whisker fabrication [11]

Transfer printing is a printing method that the patterned surface concave structure or convex structure transferred to the receptor substrate through a non-patterned stamp. The basic principle is to use the different viscosity of the printing layer relative to the stamp and the substrate to achieve pattern transfer [162–165]. There are two types of transfer printing: direct transfer printing and indirect transfer printing. In the fabricate of flexible temperature sensors, the latter often used, that is, the use of a pre-printed patterned film to transfer to the receptor substrate. In the previous review, many examples of organic materials are sensitive layers mentioned, but few inorganic materials are used as temperature-sensitive materials. It is worth noting that Liao et al. [103] reported a high-sensitivity temperature/mechanical dual-parameter sensor containing inorganic thermal material vanadium dioxide (VO_2_), which based on PET/vanadium dioxide fabricated by transfer printing technology (VO_2_)/PDMS multilayer structure. The VO_2_ layer deposited by polymer-assisted deposition (PAD) technology is etched and attached to the PDMS film. After stretching, nano-type spider web cracks formed, and then the layer press to the flexible PET substrate. It can detect the temperature in the range of 270–320 K. The temperature sensing performance with a resolution of 0.1 K attributed to the high TCR of the VO_2_ material. The collected temperature signal and the mechanical signal are separated through the algorithm's difference to achieve simultaneous monitoring the effect of temperature and mechanical changes. In order to solve the problem of the flexible temperature sensor's insufficient stretchability, Yu et al. [99], based on transfer printing technology, invented a flexible device that can maintain the sensor performance even when the flexible device is stretched or compressed by 30%. They used the Au/Cr layer as a thermistor to patterned on SOI through standard photolithography technology. The peeled heat-sensitive layer adhered through a flexible PDMS stamp, and then transferred and printed on a pre-stretched flexible PDMS substrate to release the substrate. The fabricating process of forming a stretchable flexible temperature sensor, as shown in the Fig. 7a.

Inkjet printing is an accurate, fast, and reproducible thin-film fabricating technology, which has been widely used in sensor development. Compared with other printing methods, inkjet printing has the advantages of convenience, flexibility, rapidity, low cost, compatibility, accuracy, etc. [166–168]. The patterns of inkjet printing need to be post-processed (drying, curing, sintering, etc.) to be fully formed. Improve the performance of printed patterns by converting ink nanoparticles into continuous materials. The properties of the surface tension and viscosity of the ink during the printing process, the quality of the printed pattern also places high requirements on the performance of inkjet equipment [97]. Under the condition of a specific size of the substrate, the conductive track's length formed as long as possible, and the thickness, width, and spacing of the track are reasonable. Repeated experiments obtain the ejection coefficient of the inkjet system. For example, Dankoco et al. [102] used the ink printing method deposit a composite ink with silver as the main component on the PET film to make a flexible and bendable temperature sensor. The circuit on the substrate is clear and smooth, and the ink drops are consistent, which used to measure human body temperature. The picture shows the fabricating process of the extremely sensitive and transparent multifunctional electronic skin sensor matrix developed by Oh et al. [108] The flexible array has the function of monitoring temperature, humidity, and strain. It can feel sensations, such as breathing and touch. GO and rGO, which used as humidity and temperature sensing materials, are sprayed on the PDMS substrate of the graphene circuit grown by the CVD method through inkjet printing technology through a mask. The two sensors are horizontally and vertically aligned, and the temperature sensor is on the bottom layer (as shown in Fig. 7b). After cross-lamination, a PDMS/graphene pressure strain sensor is formed. As a multifunctional flexible sensor, it can collect at the same time but independently respond to a single signal. Inkjet-printed graphene is seven orders of magnitude higher than CVD-grown graphene. The performance advantages reflected in many articles, and some research results are better than CVD-made graphite products. Inkjet printing and screen printing are both rapid and low-cost technical means to realize large-area sensor fabricating. Zirkl et al. [169] combined the two rapid fabricating technologies to create a fully printed flexible sensor array that uses multiple screens. In printing and inkjet printing, only five functional inks used to easily integrate multiple functional electronic components (including pressure and temperature-sensitive sensors, electrochromic displays, and organic transistors) on the same flexible substrate (in Fig. 7c). Because the fabricating speed and low cost of the process can also be applied to the smart sensor network using the roll-to-roll (R2R) fabricating process in the future. The development of a disposable electronic skin system (EES) is particularly critical. Similar to the previous example. Vuorinen et al. [56] introduced a temperature sensor similar to a band-aid after inkjet printing. The sensor uses graphene/PEDOT: PSS composite ink and the printing done on PU material suitable for skin. In particular, in addition to being able to achieve a sensitivity higher than 0.06% $^{ \circ } {\text{C}}^{ - 1}$, they used inkjet printers to perform serpentine patterned inkjet printing between the silver screen printed with a high resolution (1270 dpi) improved the lack of inkjet printing for printing complex graphics such as snakes. With the use and research of inkjet technology in flexible electronics, the technology for controlling nozzles and inks is also improving. Compared with the speedy fabricating process of screen printing, inkjet printing needs to go through a debugging process, and the printing speed is not as good as screen printing. Also, the small number of nozzles running simultaneously and the high nozzle failure rate limit the inkjet printing fabricating technology to laboratories, and the large-area fabricating requirements of industrial production cannot achieve.

**Fig. 8:**
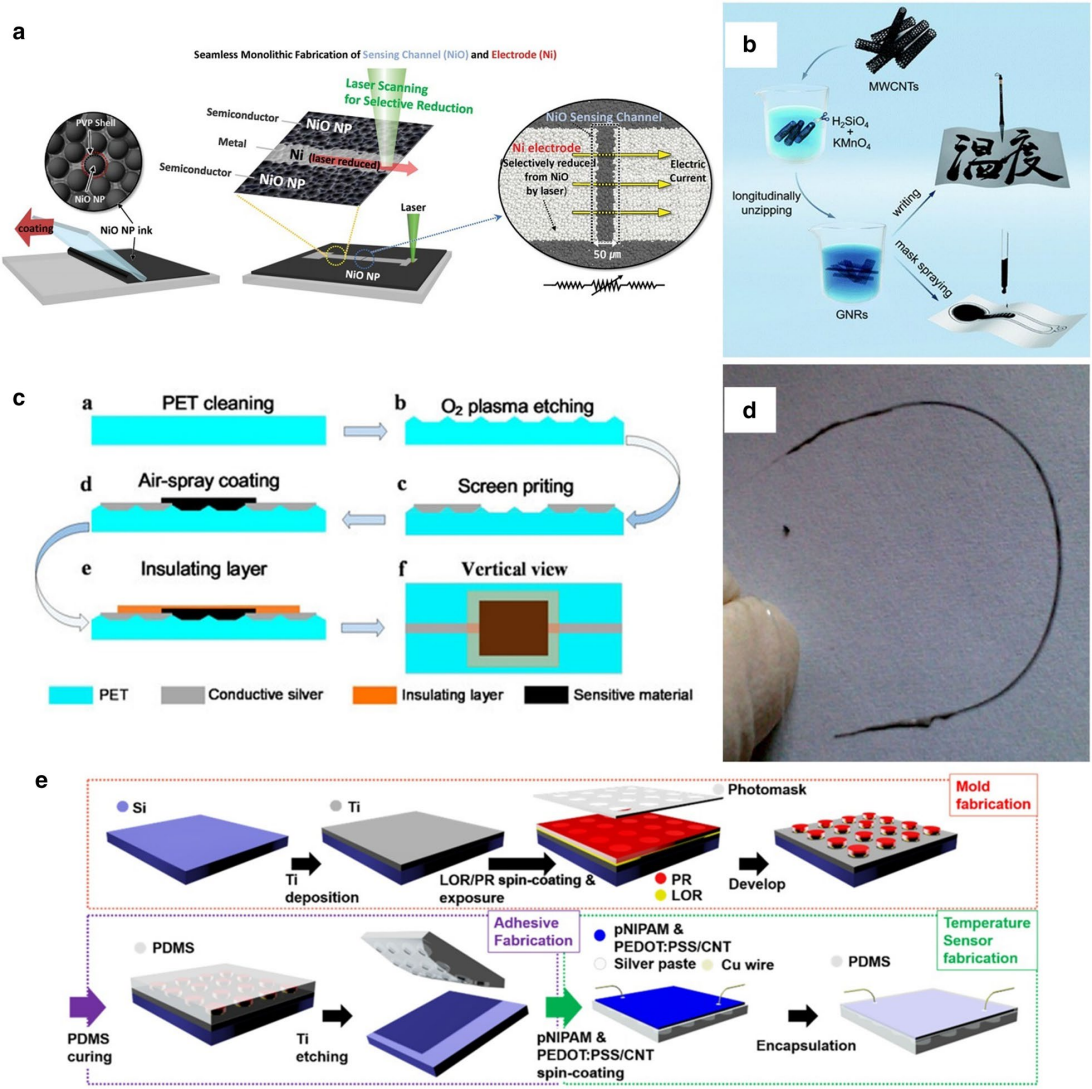
Commonly used fabricating methods. **a** Schematic of the m-LRS process [170]. **b** Schematic diagrams showing fabrication process of the paper-based GNRs sensors [171]. **c** Fabrication process of the temperature sensors [87]. **d** Flexible temperature sensor on yarn, contacts made of polymer conductive paste [111]. **e** Fabrication process for the mold, adhesive, and temperature sensor [108]

Laser direct writing (LDW) technology uses calculations to design pre-designed patterns. It directly uses laser beam ablation without masking and vacuums deposition. It can directly complete pattern transfer on the surface of the substrate material, with good spatial selectivity and high direct writing speed and processing accuracy, short cycle, high material utilization, and low pollution. Compared with traditional temperature thermistors that require high temperature and a variety of complex processes to activate the sensing function, LDW can achieve selective annealing on a predetermined pattern. A novel integral laser induced by Shin et al. [170] The laser-induced reduction sintering (m-LRS) fabricating scheme can also reduce metal oxide NPs during the annealing process. Scratch NiO NPs ink on a fragile PET substrate, and use m-LRS technology to directly reduce NiO to pattern a linear Ni electrode to form a planar Ni–NiO–Ni heterostructure (as shown in Fig. 8a). The unique method of fabricating a complete flexible temperature sensor system composed of Ni electrodes and NiO sensing channels from a single material NiO NPs provides a new idea for the rapid fabricating of flexible temperature sensors. Different from the previously designed and fabricated fully-printed sensor arrays, Harada et al. [11] proposed a more direct method of mass-fabricating flexible temperature sensors based on previous research. The flexible temperature sensing composite ink printed on the substrate, using the laser for etching away the excess part directly, leaves the designed pattern on the substrate and completes the fabricate of the temperature sensor array. Cut the base to imitate the animal's whiskers to create an artificial electronic whisker (e-whisker) structure (Fig. 7d), strain sensor formed by laminated screen printing. Bionic sensor arrays can scan and perceive three-dimensional objects by using structural advantages.

Under the premise of realizing low-cost and large-area fabricating, disposable intelligent monitoring equipment not only allows monitoring behavior to use anytime and anywhere but also ensures the safety and hygiene of the flexible monitoring equipment. Gong et al. [171] proposed a pen-on-paper fabricating method that uses a brush write or a mask to graphene nanoribbon (GNR) conductive ink dripped between the carbon nanotube electrodes on the paper base. Shown in Fig. 8b. The flexible temperature sensor fabricated after encapsulation with transparent tape. In their research, GNRs have excellent sensing performance and meet the requirements for body temperature monitoring. The fabricating method provides reliable support for the application of fast, large-area disposable flexible equipment.

#### Other Commonly Used Fabricating Methods

Spin-coating the base layer, active layer, and encapsulation layer by layer is a common way to fabricate flexible temperature sensors by the liquid phase method. Spin coating technology often used for substrate fabricating and packaging protection. For solution-like sensitive materials, the spin coating used. The coating can not only significantly improve production efficiency but also has excellent potential for large-area fabricating. Kim et al. [172] demonstrated the fabricating process of an organic field-effect transistor (OFET) array, using a spin-coating process to design a microstructure on a mold with a crystallized P(VDF-TrFE) material as a gate dielectric and other materials. Material packaging forms OFETs, and the introduction of microstructures enhances the sensing performance. The fabricating process of the bionic structure's [108] flexible temperature sensor fabricated. The adhesive layer of the bionic octopus foot sucker structure is fabricated by a spin coating process, combined with photolithography peeling technology and inverted mold technology (in Fig. 8e). It has an excellent temperature monitoring effect, and the materials used also perform well in terms of human biocompatibility. Liu et al. [87] After surface ionization treatment on the substrate, the flexible electrode was screen-printed, and then the sensitive layer rGO was connected to the flexible wire through the manufacturing method of air-spray coating, and the main structure of the temperature-sensitive flexible sensor was formed after packaging (see in Fig. 8c). It is possible to make robot skins. Huang et al. [68] fabricated a temperature sensor with flexibility, high resolution, and high repeatability in the temperature range of 25–42 °C. They dropped the PEO1500/PVDF/Gr composite solution after stirring and ultrasonic treatment on a polyimide flexible substrate. It was spin-coated at a certain speed to form a layer. It packaged it into a temperature sensor, which verified the medical body temperature transmission sense of possibility and feasibility. Spin-coating technology as an effective fabricating process for wearable devices has also introduced into Wu et al. research [173]. They designed an organic thin-film transistor with heat-sensing ability. Cleverly through the unique three-arm three-dimensional composite polylactide, polylactic acid (tascPLA) solution is spin-coated on the Au gate electrode evaporated on the Si substrate. Then the Au source and drain are deposited on it by a thermal evaporation method. The composite film containing tascPLA also serves as the gate of the OFET dielectric and substrate materials. In flexible electronics, there are not a few that use fabric as the carrier of the sensing element. The dip-coating of conductive yarn/fabric in conductive ink is the most commonly used coating technology for flexible sensors. The development of conductive fabrics provides a reasonable prerequisite for the application of smart fabrics. Sibinski et al. [111] developed a temperature sensor that monitors the temperature range of 30–45 °C. Moreover, it currently realizes filamentary miniaturization on a single yarn. In Fig. 8d shown, the PVDF monofilament fiber is coated with PMMA polymer compound mixed with multi-walled carbon nanotubes (MWCNTs) as a heat-sensitive layer by dip coating technology has good temperature sensitivity and extremely high repeatability. It is used as a fabric easily integrated into knit clothing. Another method, based on the molding properties of organic materials, is also used to fabricate flexible sensor membrane structures or to fabricate samples for testing sensing performance. The fabricating process of the thermoelectric nanogenerator (TENG) studied by Yang et al. [107] The drop-casting film method, after comparison, discards the PEDOT: PSS material, which has a weak drop-casting effect and is easy to break, not only the mixture of Te-nanowires and P3HT polymer in chlorobenzene solution is drop-casted on the flexible Kapton substrate to make a composite film, the composite material is also cast on the white fabric cloth, which also has apparent temperature sensing ability and thermoelectric performance, which can be used in wearable heat collectors. Rich and diverse fabricating methods lay the foundation for the development of flexible electronics. Under the requirements of large-scale and large-scale production, suitable fabricating technology is also continually developing. In the future, perfect wearable flexible devices are expected.

## Results and Discussion

### Key Parameters of Flexible Temperature Sensor

The flexible temperature sensor can be attached to the human body to monitor the subtle temperature changes on the surface of the human body or real-time temperature monitoring of specific parts, and even the temperature of the core or tissues and organs in the body. They respond to temperature changes by changing the resistance and output the temperature changes as electrical signals. With the increasing demand for flexible electronic devices, the disadvantages of traditional temperature sensors, such as poor scalability, inability to carry, and poor real-time performance, are becoming increasingly unsuitable for flexible wearable devices. Today flexible temperature sensors are required to have high performance, such as high sensitivity, fast response time, reasonable test range, high precision, and high durability to realize the monitoring function better.

#### Sensitivity

Sensor sensitivity refers to the ratio of the change ∆*y* of the system response under static conditions to the corresponding input change ∆*x*, that is, the ratio of the dimensions of output and input. When the sensor output and input dimensions are the same, the sensitivity can be understood as the magnification [21, 174, 175]. The temperature coefficient of resistance TCR (TCR, in $^{ \circ } {\text{C}}^{ - 1}$) of the common resistance type flexible temperature sensor is expressed in the following expression, $\Delta R/R_{0} = s\left( {T - T_{0} } \right)$, is the relative resistance change ($\Delta R/R_{0}$) as a function of temperature, where $s$ represents TCR. If the sensor output and input show a linear relationship, the sensitivity is constant [83]. Otherwise, it will change with the input quantity. Generally speaking, by increasing the sensitivity, higher measurement accuracy can be obtained. Most flexible sensors used for body temperature monitoring only pay attention to temperature changes within 10 °C and therefore require high-temperature sensitivity to capture relatively small temperature changes. Here, several typical methods and concepts for improving the sensitivity of flexible temperature sensors are summarized to provide a favorable reference for further improvements.

**Fig. 9:**
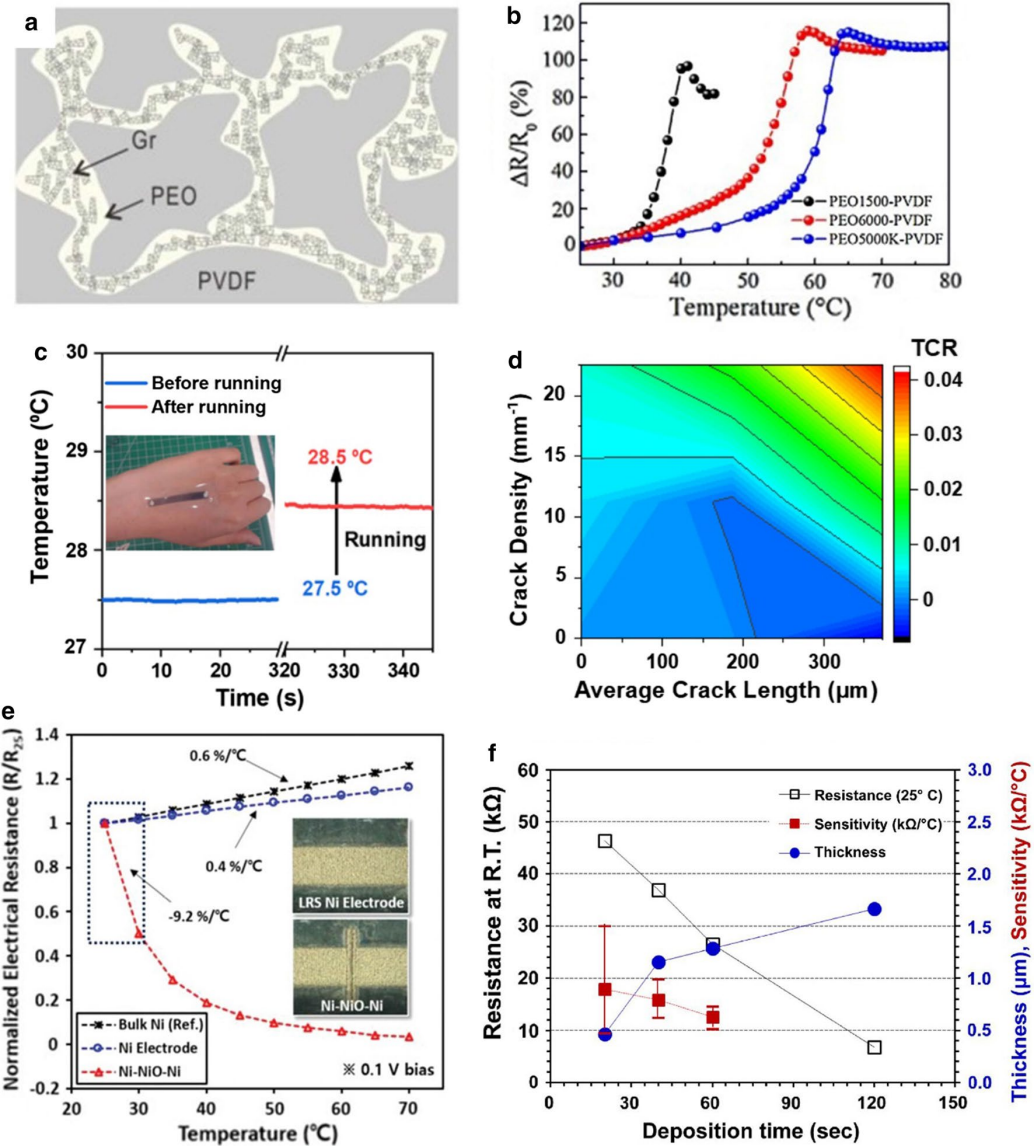
Sensitivity of flexible temperature sensor. **a** Images of the PEO1500/PVDF/Gr at room temperature. **b** Resistance curves of PEO1500/PVDF/Gr, PEO6000/PVDF/Gr and PEO5000K/PVDF/Gr composites versus temperature [68]. **c** The temperature measurement results before and after exercise, the illustration shows the flexible temperature sensor attached to the back of the hand. **d** Heatmap as functions of average crack length and crack density for TCR valuses from all developed sensors [176]. **e** PTC and NTC characteristics of the m-LRS processed Ni electrode and Ni–NiO–Ni structure. Inset digital images are m-LRS processed Ni electrode (upper) and Ni–NiO–Ni structure (lower) [170]. **f** The results of the deposited film thickness (blue circle), resistance at room temperature (white square) and sensitivity (red square) as a function of deposition time [177]

The sensing performance of flexible temperature sensors is usually closely related to the properties of materials. One possible way to realize the development of sensitive sensors is to adopt composite materials with visible thermal performance. After temperature stimulation, the internal conductive network will change, which will lead to affect the temperature sensitivity of the device. To improve the temperature sensitivity of the active material, a strategy that converts temperature fluctuations into mechanical deformation is adapted to amplify the response of the conductive network to temperature changes. The thermosensitive material is connected to a substrate with a high positive thermal expansion coefficient to enhance thermal induction deformed. This method has applications in sensors made of graphene, graphite and graphene-nanowalls. For example, in the flexible temperature sensor designed and fabricated by Huang et al. [68], Gr establishes a conductive path in the PEO/PVDF binary composite material. In the process shown in the Fig. 9b. The temperature change will cause the PEO to transform from crystal to amorphous, and melt when the temperature is close to the melting point of PEO, the volume expansion of PEO will destroy the conductive network in the composite material, resulting in a sharp increase in resistivity-increasing the strength of the PTC, which can quickly reaction within a narrow temperature range change. Experiments show that PEO thermal performance plays a leading role in the PTC effect of the device. Another way to improve the sensitivity of a flexible temperature sensor is to use a unique structure. In recent years, to improve temperature sensing performance, various design strategies have been developed by changing the structure. Yu et al. [176] recently proposed a method based on engineering microcrack morphology to change the crack morphology of the PEDOT: PSS film on the PDMS substrate by adjusting the substrate surface roughness, acid treatment time, and pre-stretching degree to improve the temperature sensitivity of the sensor. Figure 9d shows the effect of average crack length and cracks density on temperature sensitivity. The result is that the longer the crack length, the higher the crack density, and the higher the temperature sensitivity. It is proved that the micro-crack structure plays a vital role in the temperature sensitivity of the sensor. It is verified that obtaining a high-density micro-crack structure is the key to obtaining the high-temperature sensitivity of the sensor. High density and high length directly correspond to higher temperature sensitivity. The fabricating process of flexible electronic equipment plays a vital role in the production of the device and can effectively improve the sensitivity of the flexible temperature sensor. Just as Shin et al. [170] used a rapid overall laser-induced reduction sintering (m-LRS) method for fabricating Ni/NiO flexible temperature sensor is different from the inkjet printing method. They directly reduce and sinter the Ni electrode on the NiO layer, and form a high-quality overall contact between the metal electrode (Ni) and the temperature-sensitive material (NiO). Ni–NiO–Ni heterogeneous temperature sensor shows higher temperature sensitivity than other sensors of the same type (Fig. 9e). Raman spectroscopy and X-ray diffraction (XRD) measurements show that the superior sensitivity comes from the unique thermal activation mechanism of the m-LRS process. Besides, since flexible temperature sensors are mostly touch-sensitive, it is inferred that the film's thickness affects the sensitivity. The study of Lee et al. [177] verified the effect of the thickness of the highly sensitive paper base on the sensitivity, they use a simple dipping fabricating method to deposit sensitive materials on the printing paper. Dipping time will affect the thickness of the film. The Fig. 9f also shows the different performance of sensitivity due to different thicknesses. It should be noted that the higher the sensitivity, the narrower the measurement range and, worse, the stability. Therefore, it is necessary to pay attention to increasing stability and accuracy while improving sensitivity.

#### Other Parameters of Flexible Temperature Sensor

The sensing range of a flexible temperature sensor is an important parameter, which refers to the minimum and maximum temperature that can be detected. In this article, we are only interested in the measurement range (30–45 °C) suitable for body temperature monitoring. Another vital performance parameter, response time, is generally defined as the time consumed by the temperature sensor from applying temperature stimulation to generating a stable signal output. Some documents also define the temperature sensor's response time by the time the sensor temperature rises from $T_{{{\text{sensor}}}}$ to 90% of the temperature rise ($T_{0}$) [145]. It is related to the thermal response of the active material itself and reflects the rapid response ability of the temperature sensor to temperature. In terms of applications, such as real-time human body health-monitoring products and wearable artificial intelligent elements with an instant response, all have a shorter response time.

The sensor ability to sense the smallest amount of change to be measured is defined as resolution. In other words, the input quantity starts to change from a non-zero value. At this time, if the input change value does not exceed an absolute value, and the sensor's output does not change, it means that the sensor cannot distinguish the change of the input quantity. Accuracy refers to the ratio of the value of plus or minus three standard deviations near the real value to the range, the maximum difference between the measured value and the real value, and the degree of dispersion of the measured value. For measuring instruments, accuracy is a qualitative concept and generally does not require numerical expression. Because the normal fluctuations of human body temperature within a day are small, high resolution or high accuracy is significant for flexible temperature sensors for body temperature monitoring, determining the broader application of flexible temperature sensors.

Repeatability is the degree of inconsistency of the measurement results for the same excitation quantity when the measurement system performs multiple (more than 3) measurements on the full range in the same direction under the same working conditions. In a flexible temperature sensor, durability means that it maintains a stable sensing function and a complete device capability under a long-term use environment. A flexible temperature sensor with high durability and high repeatability can meet the basic requirements of long-term stable use. Linearity usually defines as the degree of deviation between the actual input–output relationship curve and the ideal fitting curve, usually expressed as a percentage. Therefore, in the linear range, the output signal will be more accurate and reliable. High linearity is also conducive to the input–output signal calibration process and subsequent data optimization processing [58]. The demand for the development of flexible temperature sensors with high linearity is also increasing.

### Applications

The ability to live organisms to respond accurately and quickly to external environmental stimuli is an essential feature of life. The induction of temperature allows humans to predict dangers and respond to diseases. Abnormal changes in body temperature often play a crucial reminder and help for early prevention [179]. With the research and development of flexible electronic devices, such as electronic skins, smart health monitoring systems, smart textiles, and biomedical equipment, sensors with the multi-functional signal acquisition are necessary [180, 181]. Among them, the flexible temperature sensor is an indispensable and vital part of medical and health applications [96]. In the past few years, the progress of new materials, new fabricating technologies, and unique sensing methods have provided an essential reference and a solid foundation for the development of a new type of skin-like flexible temperature sensor. In these sensors, some basic performance parameters such as sensitivity, resolution, and response time even exceed natural skin. Although, after a lot of basic and applied research, research results can be transformed from the laboratory to real-world use. However, there are still many challenges waiting to be solved in practical applications. In the following content, we will summarize recent flexible temperature sensors' results in flexible electronics applications with unique, excellent, and practical application examples.

**Fig. 10:**
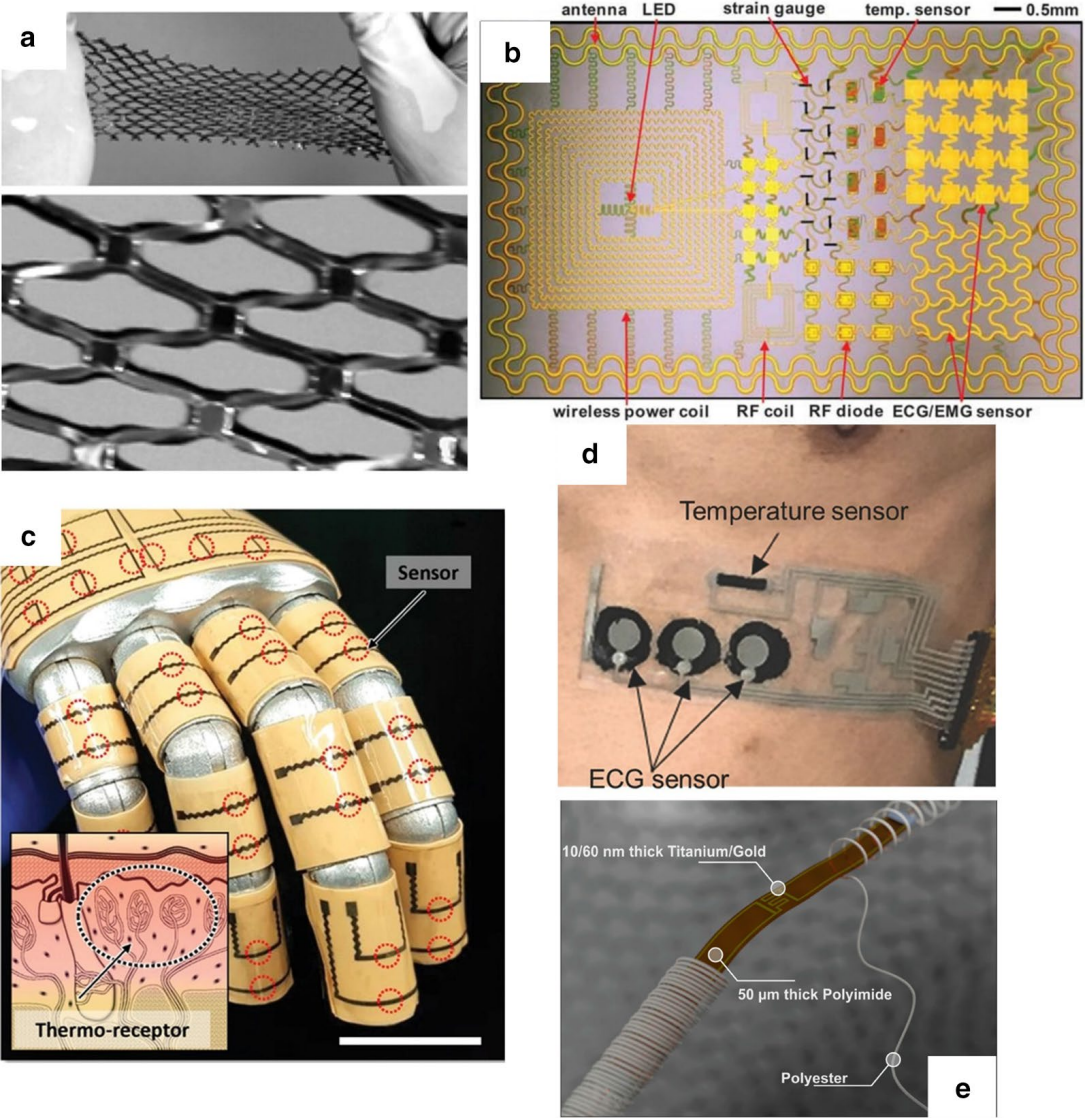
Application demonstration of flexible temperature sensor. **a** A plastic film with organic transistors and pressure-sensitive rubber is processed mechanically to form a unique net-shaped structure, which makes a film device extendable by 25%. A magnified view of extended net-structures is also shown [187]. **b** Image of a demonstration platform for multifunctional electronics with physical properties matched to the epidermis [155]. **c** Model hand covered with the temperature-sensitive artificial skin with enlarged illustration of skin thermos-receptors [170]. **d** Photo of the fabricated device [184]. **e** Concept of the flexible temperature sensor embedded within the fibres of a textile yarn [185]

Flexible sensors have the characteristics of large-area deformability, lightweight, and portability, which can realize functions that traditional sensors cannot. Electronic skin integrates various sensors and conductors on a flexible substrate to form a highly flexible and elastic sensor similar to the skin. It converts external stimuli such as pressure, temperature, humidity, and hardness into electrical signals and transmits them to the computer for processing, even can recognize regular objects [14]. An electronic skin with similar functions is a necessary feature of future robots to achieve perception in an unstructured environment. The electronic skin enables the robot to perceive changes in the external environment as sharply as a real person. Although the principle of electronic skin is simple, there are still challenges in covering the surface of the robot with electronic skin. The use of the electronic skin determines, that is, maintaining the device's integrity while maintaining the sensing performance during mechanical deformation. The ideal method of flexible electronic skin is patterned fabricating. Using solution materials, directly deposited on the substrate through printing technology to form patterns, and achieve roll-to-roll (R2R) large-area fabricating under normal temperature and pressure so that the skin has the advantages of considerable size, high yield, low production cost, and environmental protection. Someya et al. [182] have developed an electronic mesh skin with flexibility, large area, integrated temperature, and pressure-sensing capabilities, as shown in the figure. The stretchable electronic skin has multiple heats, and pressure sensors distributed at the nodes and read data through OTFT. Among them, the thermal sensor array is based on organic diodes, prepared on polyethylene naphthalate (PEN) coated with indium tin oxide (ITO) on the surface. The thermal film is mechanically cut by a numerical control cutting machine, and the R2R process prepares the network structure, and then the network film is combined by lamination to complete the preparation of the thermal sensor array. The parylene protective layer is placed on the organic semiconductor layer to act as a flexible gas barrier layer, extending the device's durability from days to weeks and avoiding mechanical damage to transistors and diodes during testing. When the sensor is in its original state, the space is square, and it becomes a rhombus after being stretched, and it can still maintain excellent electrical characteristics when stretched 25% (shown in Fig. 10a). The establishment of the mesh structure expands the use of electronic skin. The distributed structure of multiple parameters and multiple nodes also makes it possible to monitor irregularly shaped objects. A kind of epidermal electronics proposed by Kim et al. [53] refers to ultra-thin flexible electronic devices fixed on the skin's surface (in Fig. 10b). Only through van der waals force can it fit perfectly with the skin and sense the temperature, strain, and dynamic response. Potential applications include physiological state detection (electroencephalogram, electrocardiogram, electromyography), wound detection or treatment, biological/chemical perception, human–computer interaction interface, wireless communication, etcetera. Integrating all devices in the measurement device in a completely different way integrates a variety of functional sensors (such as temperature, strain, electrophysiology), micro-scale light emitting diodes (LEDs), active/passive circuit units (transistors, diodes, resistors), wireless power supply coils, wireless radio frequency communication devices (high-frequency inductors, capacitors, oscillator, and antenna). Skin electronics has the characteristics of ultra-thin, low elastic modulus, lightweight, and ductility. The device is fabricated in the form of winding-shaped filament nanowires or micro-nano thin films, enabling the system to withstand more significant elastic deformation. It can be easily transferred to the skin surface through the soft-touch process, just like a tattoo sticker. Although the electronic skin has wealthy functions, the resonance frequency will drift when the strain exceeds 12%. Besides, the durability of ultra-thin flexible devices also requires attention. The shortcomings mentioned above need to be considered when designing and fabricating future electronic skin systems.

The development of a wearable health monitoring system is for collecting human body thermal parameters: body temperature, epidermal temperature, heat flow [183], etcetera. For observation and inference such as metabolism, fever, disease infection, skin healing, thermal adaptation (implants, prostheses), etc. Compared with the limitations of traditional monitoring systems in the use cases. Flexible wearable temperature monitoring systems have demonstrated more flexible applications and excellent thermal parameter monitoring effects in recent studies—the m-LRS NiO temperature sensor system fabricated by the Shin et al. team that we introduced earlier. In an experiment to verify the potential of its system as an electronic skin application. As shown in the Fig. 10c, three side-by-side temperature sensors attached to the robot finger can make an accurate flow rate of hot or cold water flowing in the microfluidic pipe, flow direction, and temperature response. Besides, it has a high-resolution capability for the temperature increase caused by infrared heat radiation. Moreover, the high-curvature fit feature allows the temperature sensor system also to monitor breathing to provide early warning of abnormal respiratory symptoms caused by disease or poisoning. Experiments on breathing changes before and after exercise have verified the system's effectiveness in monitoring small changes in human breathing and temperature. The holistic fabricating technology they put forward provides useful help for the large-scale development of the healthcare system and electronic skin. Webb et al. [145] reported two types of flexible, ultra-thin, and sensitive temperature sensors/actuators with different structures, namely, a TCR sensor array with a serpentine network formed by Cr/Au fabricated by microlithography technology and a TCR sensor array after deposition. Corrosion nano PIN sensor, the two sensors have high sensitivity, rapid response, and high precision. Non-invasive monitoring of the subtle changes in skin surface temperature caused by changes in human blood flow caused by external stimuli can be realized. By accurately measuring skin thermal conductivity and monitoring skin moisture, it has practical application value for health management such as blood sugar monitoring, drug delivery and absorption, and wound or malignant tumor changes. For a flexible sensor system that needs to monitor health status based on vital signals, easy fabricating, secure attachment, and strong biological adaptability are essential. As Fig. 10d shown, Yamamoto et al. [184] developed a gel-free viscous electrode that is more suitable for electrocardiogram (ECG) sensors. A temperature sensor fabricated by patterned printing is assembled to form a flexible sensor system that can monitor the occurrence of dehydration or heatstroke symptoms. The use of biocompatible materials solves the problem that traditional gels cannot be attached to the human body for a long time. The open, flexible sensing system platform can also add various sensors to develop a broader range of applications. The temperature and heat transfer properties of the skin can be characterized as critical information for clinical medicine and basic research on skin physiology. In terms of thermal adaptation, it is crucial to monitor and predict the residual limb's skin temperature. For example, the prosthesis gaps create a warm and humid micro-environment, which promotes the growth of bacteria and causes inflammation of the skin. Local skin temperature monitoring uses flexible temperature sensors hidden in daily textiles. Due to its high consistency, it is very suitable for non-existent monitoring of skin temperature, which significantly benefits patients and medical staff. Lugoda et al. [185] used different industrial yarn fabricating processes to integrate flexible temperature sensors into fabrics, and studied the sensing effect of embedding flexible temperature sensors in textile yarns. The bending resistance and repeatability of the temperature sensor embedded in the yarn are verified. The elongated sensor is made by patterning a Ti/Au layer deposited on a PI film (see in Fig. 10e). Experiments with the temperature sensor embedded in the armband have verified that this sensor yarn can be used to fabricate intelligent temperature-measuring textile garments. Smart temperature measuring textiles can be used for thermal adaptation monitoring applications such as the detection of thermal discomfort in prostheses, socks for early prediction of diabetic foot ulcers [186], textiles that continuously measure the body temperature of babies, and bandages for monitoring wound infections. The use of industrial yarn fabricating technology to integrate flexible temperature sensors makes large-scale smart textile fabricating possible.

**Fig. 11:**
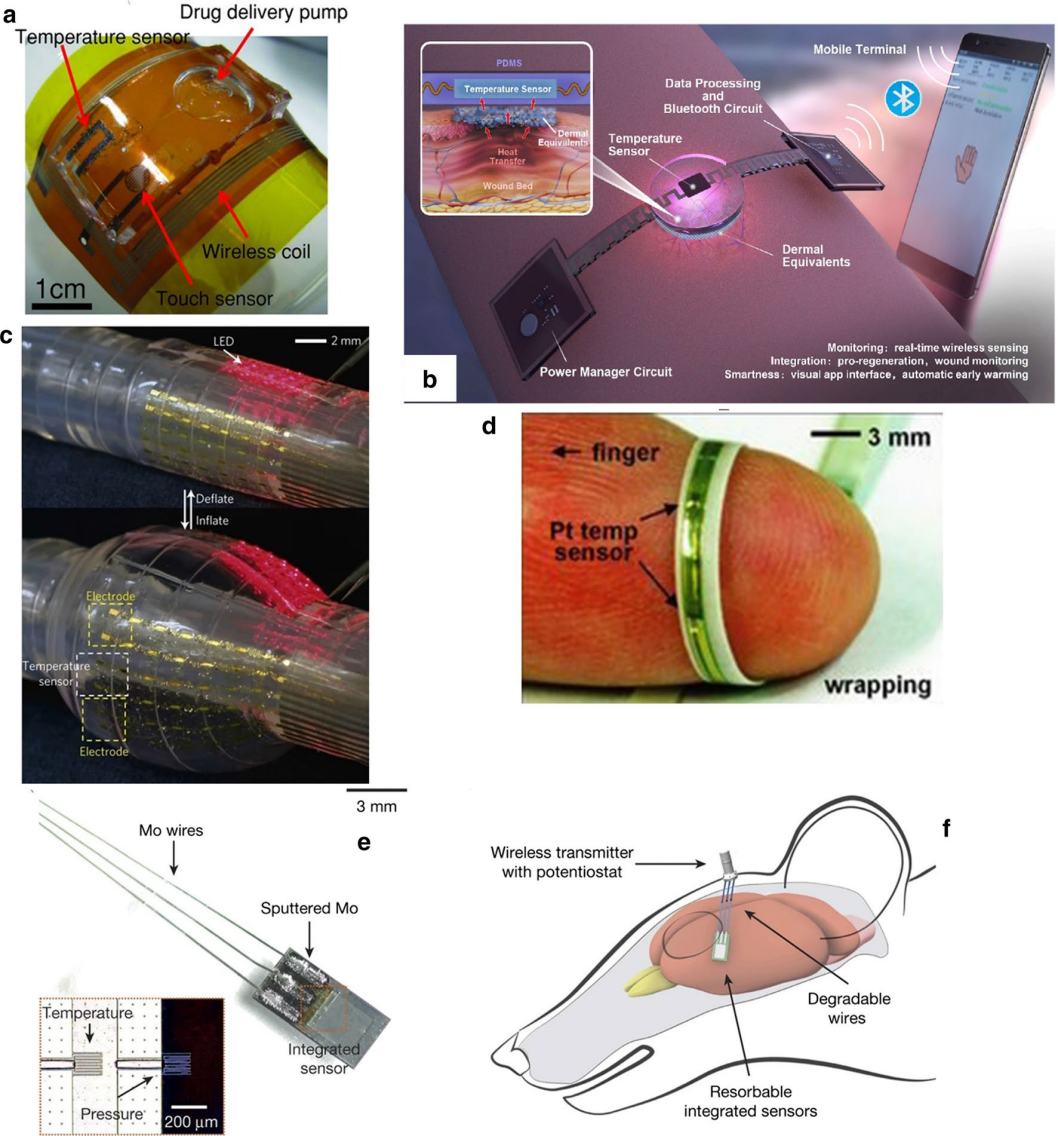
Application demonstration of flexible temperature sensor. **a** Photos of the smart bandage integrated with touch and temperature sensors, a wireless coil, and a DDP [133]. **b** The application scenario of FWHS. The FWTSD with a Band-Aid shape tightly contacts with the wound site. Temperature variation is detected by temperature sensor [189]. **c** Optical image of a multifunctional balloon catheter in deflated and inflated states [191]. **d** Optimized mechanical structure shown in a schematic illustration when wrapping [53]. **e** Image of bioresorbable pressure and temperature sensors integrated with dissolvable metal interconnects and wires. **f** Diagram of a bioresorbable sensor system in the in a rat's skull [193]

With the development of flexible electronics, the application of multifunctional flexible wearable devices in health care, smart monitoring equipment, human–computer interaction/combination, smart robots, and other related fields have become more and more extensive. Flexible temperature sensors have strong practicability in flexible wearable electronic devices, especially for monitoring human health, which is inseparable from accurate temperature control. The introduction of the wireless transmission function allows people to analyze and utilize the temperature sensor's measurement results. Honda et al. [133] proposed a wearable human–computer interaction device, a patterned "smart bandage" with drug delivery function. The device uses low-cost patterned printing technology to integrate temperature sensors, MEMS-structured capacitive tactile sensors, and wireless coils on a flexible substrate. Figure 11a shows the physical object of the flexible device on the arm. A drug delivery pump (DDP) made by soft lithography and a microchannel for drug discharge can be added to supply drugs to wounds or internal parts to improve the wearer's health. As a wearable interactive humanized health monitoring wireless device application, it will play a positive role in future wearable electronic applications. Intelligent wound patch: wounds after trauma are infected and difficult to heal. Under traditional wound dressings, the healing process of wounds is often unpredictable, and satisfactory repair results cannot be obtained. In wound infection or healing, temperature changes different from the normal body temperature will occur. Therefore, monitoring the subtle temperature changes at the wound is quite necessary for ideal wound healing [188]. To eliminate the "black box" state in the wound healing process and grasp the wound's situation in real-time, Lou et al. [189] proposed a transparent and soft, closed-loop wound healing system to promote wound healing real-time monitoring of the wound. The flexible wound healing system (FWHS) and flexible wound temperature sensing device (FWTSD) likes the form of band-aids, their flexible device in the shape of a double-layered band-aid can be directly attached to the wound. The upper layer is a flexible temperature sensing layer composed of temperature sensors, power management circuits, and data processing circuits. The lower layer is a collagen-chitosan dermis equivalent for skin regeneration. A customized software application (app) installed on the smartphone receives data from the sensing layer, displaying and analyzing wound temperature in real-time (Fig. 11b). The system has high sensitivity and stability, good ductility, reliability, and biocompatibility. In addition to monitoring the normal wound regeneration process, in the biological experiments, timely warning of severe wound infection was achieved. A smart wound healing system with growth promotion, real-time temperature monitoring, wireless transmission, and visualization that integrates wound monitoring, early warning, and on-demand intervention may occur in the future. If the wound after an injury is severely infected and is not treated in time, more serious damage to the body may occur. Timely monitoring of wound conditions and rapid, effective diagnosis and treatment are urgent needs to reduce the occurrence and aggravation of wound infection complications. In another study, Pang et al. [190] proposed a flexible smart bandage with a double-layer structure with high sensitivity, good durability, and remote control. The upper layer is packaged in PDMS connected and integrated through a serpentine wire. The temperature sensor, ultraviolet (UV) light-emitting diode, power supply, and signal processing circuit are designed, and the lower layer is designed with a UV-responsive antibacterial hydrogel. When the temperature sensor detects an abnormal temperature exceeding the threshold, it will wirelessly transmit the signal to the smart device. Moreover, control the in-situ ultraviolet radiation through the terminal application to release antibiotics from the underlying hydrogel and apply it to the wound to achieve early infection diagnosis and processing. The system can monitor wound status in real-time, diagnose accurately, and treat on-demand. The research of this intelligent trauma diagnosis system provides new strategies and diagnostic and treatment ideas for the prevention and treatment of traumatic diseases such as wound treatment and diabetic ulcers. The popularization and application of such systems also have significant prospects. The development of advanced surgical tools is an important measure to improve human health. The advancement of flexible electronics provides reliable support for the flexibility and miniaturization of clinical medical instruments. Simultaneously, the function of flexible devices is enriched, allowing clinical instruments to perform multiple operations on diseased parts in a short period, reducing the risk of surgery. In Fig. 11c,d, Kim et al. [191] invented a direct integration of a multifunctional element group, including a flexible temperature sensor array with the elastic film of a traditional balloon catheter, which provides a variety of functions for clinical applications. The use of this balloon catheter in live animal experiments verified the instrument's ability to provide key information about the depth of the lesion, contact pressure, blood flow or local temperature, and the radiofrequency electrodes on the balloon for tissue controlled local ablation. Specific use in cardiac ablation therapy. In another study, they developed two thin and bendable flexible temperature sensor applications. Used for medical sutures, one of the simplest and most widely used devices in clinical medicine. One is an ultra-thin suture line based on the integrated Au thermal actuator and silicon nanodiode temperature sensor on the biofiber membrane. The other suture line is two $1 \times 4$ Pt metal temperature sensor arrays formed between Au wires on both sides of the substrate. To achieve the purpose of helping biological wounds heal and monitoring wound recovery. It is full of challenges to build semiconductor devices and sensors and other components on a biologically adaptive platform that touches the flexible curved surface of the human body.

**Fig. 12:**
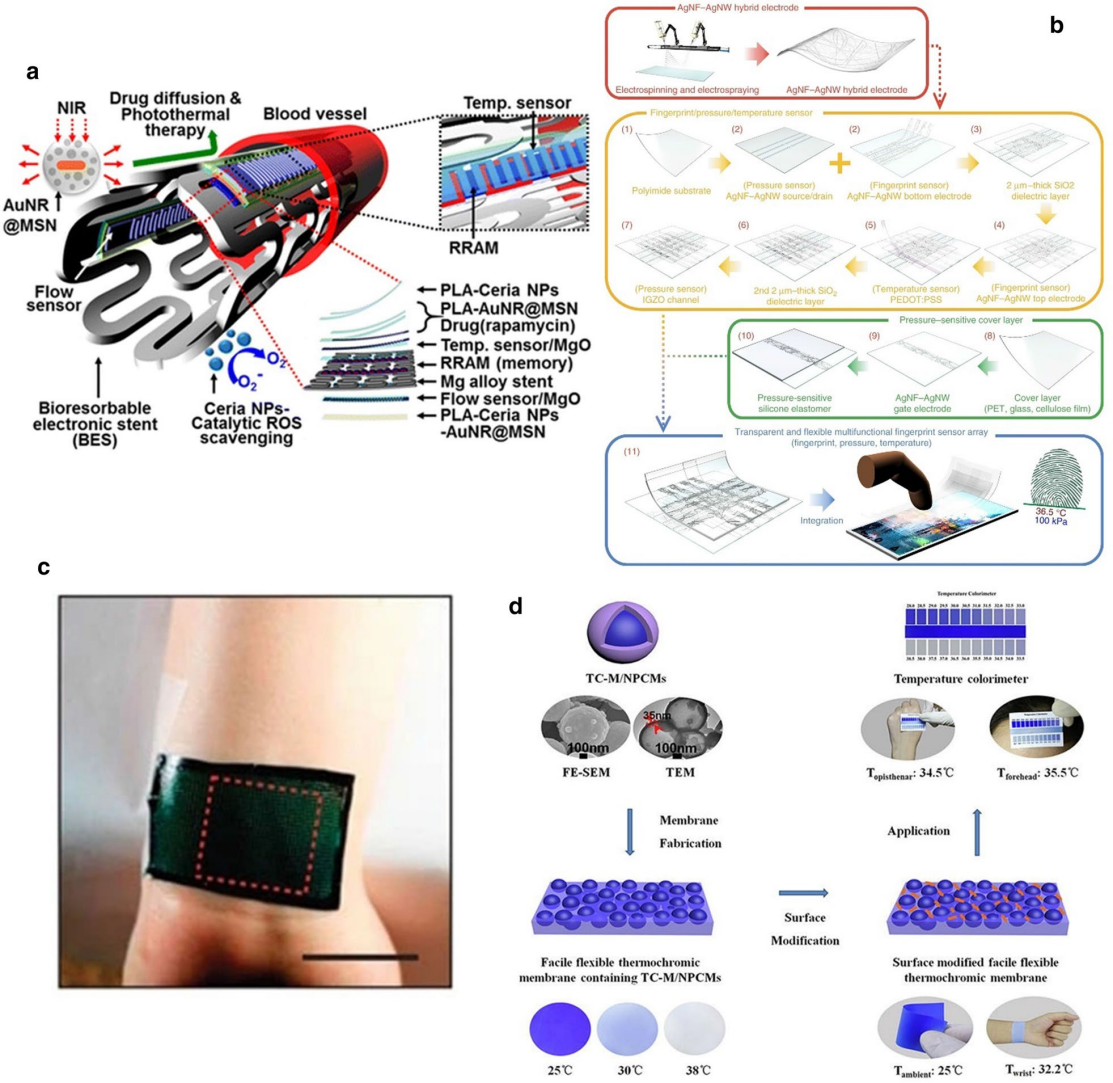
Application demonstration of flexible temperature sensor. **a** Schematic illustration of the BES (left), its top view (top right), and the layer information (bottom right). The BES includes bioresorbable temperature/flow sensors, memory modules and bioresorbable/bioinert therapeutic nanoparticles [194]. **b** The fabrication process for the multiplexed fingerprint sensor [196]. **c** Optical images of an e-TLC device on the wrist during an occlusion test (scale bar, 3 cm) [15]. **d** Facile flexible reversible thermochromic membranes based on micro/nanoencapsulated phase change materials for wearable temperature sensor [198]

As we all know, some operations will leave objects different from the body's tissues such as steel plates, stents, pacemakers, implants, etcetera, inside the body, which will cause discomfort to the body [192]. The development of absorbable devices will optimize surgical implantation equipment Post-access problems reduce the risk of surgery and the difficulty of post-wound healing. Kang et al. [193] reported a multifunctional silicon sensor. The experiment of implanting the sensor in rats' brains proved that all the materials (polylactic-glycolic acid, silicon nanomembranes (Si-NMs), nanoporous silicon, SiO_2_) constituting the sensor could be used. Naturally absorbed through hydrolysis and/or metabolism, no need to extract again. It can continuously monitor parameters such as intracranial pressure and temperature, which illustrates the performance advantages of absorbable devices for treating brain trauma. The emergence of implantable vascular stents provides strong support for the smooth progress of interventional surgery. The vascular stent can expand the blood vessel through a continuous structure in the blocked blood vessel to restore blood flow. However, the inflammation caused by traditional vascular stents in the body for a long time is still difficult to be diagnosed and cured. Son et al. [194] introduced the bioabsorbable electronic stent (BES) applications based on bioabsorbable and bioinert nanomaterials.(Fig. 11e,f) Integrated flow sensing, temperature monitoring, wireless power/data transmission, and inflammation suppression, local drug delivery, and hyperthermia device. The Mg temperature and flow sensor are composed of an adhesion layer, a fiber-shaped Mg resistance (sensing unit), and an outer encapsulation layer (MgO and polylactic acid(PLA)) (as shown in Fig. 12a). Experimental studies have shown that combining bio-absorbable electronic implants and bio-inert therapeutic nanoparticles in intravascular smart stent systems has not yet been recognized.

Temperature visualization makes the function of flexible temperature sensors more prominent. It makes up for the disadvantages of traditional infrared imaging equipment that are expensive, unfavorable for carrying, and inaccurate in measuring dynamic objects. The appearance of high-intensity focused ultrasound (HIFU [55]), smart device monitoring, and other occasions will make temperature measurement more accurate and useful [195]. An et al. [196] developed a PEDOT: PSS temperature sensor with ultra-long silver nanofibers (AgNFs) and silver nanowires (AgNWs) hybrid network as a high-performance transparent electrode, layer, and patterned fabricating, using multiplexing technology to create a capacitive flexible and transparent multifunctional sensor array (Fig. 12b). The capacitance change is 17 times higher than that of a pattern sensor that also uses ITO electrodes. In the future, the visual display of fingerprints, pressure, and temperature changes between the fingers on the flexible sensor can be realized simultaneously on smart devices. Applying in the fingerprint recognition function, the security function of smart mobile devices can improve more security. Gao et al. [15] introduced a light-emitting device that combines a colorimetric temperature indicator with wireless, patterned, and stretchable flexible electronic technology. A large-scale thermochromic liquid crystal (TLC) pixel array is formed on a thin elastic substrate in combination with colorimetric reading and radio frequency (RF) drive to map the thermal characteristics of the skin. Under the premise of non-invasiveness, the sensor system uses radio frequency signals to control local heating to survey and map skin temperature with high-temperature accuracy (50 mK) and high spatial resolution. The blood flow under the skin is evaluated by the reactive hyperemia test (see in Fig. 12c). Also, hydration analysis reflects skin health problems. The figure verifies that the device can quickly visually respond to small changes in blood flow. Reflect cardiovascular and skin health problems through hydration analysis. Like the fluoroscopy capability of an infrared camera, the device can be used for core temperature and wound healing monitoring, near-body implant device inspection, malignant tumor cancer screening, and other biomedical-related functions. The unique feature is that the flexible device can be read using a mobile phone, and it can be worn for a long time. The temperature visualization system provides a massive prospect for the description of the thermal properties of the skin. It makes efforts to provide useful indicators for determining the human health and physiological state. In another study, Kim et al. [197] provided a feasible idea using thermochromic materials to observe temperature changes directly. The difference is that their description of the temperature change does not have a specific numerical display, and can only judge the approximate temperature change range. However, as shown in Fig. 12d, He et al. optimized the accuracy of the mapping temperature of thermochromic materials, which can reflect the specific value of the temperature more accurately [198]. In future research, the development of thermochromic materials sensitive to temperature and can accurately respond to temperature will also gradually becoming one research focus. In particular, the reverse cushioning material they made shown a color similar to human skin. Besides, they used 3D printing technology to fabricate the flexible pressure sensor, simplifying the fabricating process, because the sensor structure parameters can be adjusted, showing customizable functions, and achieving the purpose of overall structural fabricating. In the future, 3D printing is the best candidate for the development of unique functional structures [199]. Because this technology can achieve the overall fabricating of the device, it simplifies the fabricating process and ensures integrated molding requirements. It will also gain enormous popularity in the field of flexible sensors.

## Conclusions

This article reviews the recent research progress of high-sensitivity flexible temperature sensors in human body temperature monitoring, heat-sensitive materials, fabricating strategies, basic performance, and applications. As a relatively stable dynamic variable in the human body, body temperature or local temperature (trauma) may have different degrees of small fluctuations (about 0.5 °C) under the influence of emotions or physiological activities. The monitoring temperature of the flexible temperature sensor needs to be comparable. Traditional infrared cameras have smaller temperature resolution (< 0.1 °C). Besides, timely and fast monitoring of body temperature is another key to breakthrough. The current temperature sensor response time can be within a few milliseconds, but there is a problem of too long reset time. How to shorten the time difference between response time and reset time will directly affect sensor monitoring's efficiency and capability. With the development of multifunctional sensors, how to avoid mutual interference between multiple signals so that the stimuli of the respective responses between the signals can independently respond without mutual interference and accurately output, which has become an urgent problem to be solved and perfected for improving sensor performance. The development of flexible temperature sensors shows us a foreseeable future. In the future, flexible temperature sensors will also achieve large-area low-cost fabricating, high sensitivity, self-supply, visualization, self-healing, biodegradability, and wireless remote sensing transmission [200]. Furthermore, other functions are integrated and put into use. Sorting the collected temperature data into the health big data platform can provide the best help and data support for human future medical diagnosis. Also, patterned micro-nano fabricating technology is a good suggestion for low-cost mass production of sensors. Based on the relatively mature process flow of the printing process, the realization of integrated multifunctional large-area flexible devices is just around the corner. The current flexible temperature sensor can achieve higher sensitivity, but some sensors do not eliminate environmental factors' interference on the temperature sensor. In the future, the flexible temperature sensor used for body temperature monitoring can make efforts to combat environmental influences. Although the flexible sensor itself can be fragile and light, it needs to be connected to the power supply circuit and the power supply, which dramatically reduces the overall flexibility. In the future, for flexible temperature sensors that monitor body temperature, with the further optimization of signal acquisition methods, real-time visual data wireless transmission can be realized under more efficient self-powered conditions, which will be a vast improvement for intelligent monitoring systems. The monitoring of body surface temperature is greatly affected by the environment, while the core temperature is relatively stable. The flexible temperature sensor used for body temperature monitoring can be attached to the body surface (forehead, arm, armpit, etcetera.) to monitor the body surface temperature and even fluctuate. The core temperature with a small range can also be measured. Non-implantable flexible sensors require more improvements in wearability, biocompatibility, and durability to meet the needs of a broader range of people and become a flexible application device available to everyone. For intrusive flexible temperature sensors, whether in the process of intrusion or during the use of the sensor, minimizing damage to the body is the primary consideration. Therefore, exploring and developing biocompatible or biodegradable sensing materials and sensors is undoubtedly an improvement direction. There will be no rejection or allergic reactions in the body due to foreign bodies. In the future, flexible temperature sensors will appear on many occasions around us. The exploration of the development of flexible temperature sensors with high performance, easy fabricating, low cost, and wide application range will continue.

## Data Availability

Not applicable.

## Abbreviations

PDMS: Polydimethylsiloxane
PI: Polyimide
PU: Polyurethane
PET: Polyethylene terephthalate
PVA: Polyvinyl alcohol
PVB: Polyvinyl butyral
CTE: Coefficient of thermal expansion
CB: Carbon black
CNTs: Carbon nanotubes
TCR: Temperature coefficient of resistance
EG: Expanded graphite
Gr: Graphite
PEO: Polyethylene oxide
PVDF: Polyvinylidene fluoride
PEDOT: PSS: Poly(3,4-ethylene dioxythiophene)-poly(styrene sulfonate)
MWCNTs: Multi-walled carbon nanotubes
SWCNTs: Single-walled carbon nanotubes
GO: Graphene oxide
rGO: Reduced graphene oxide
GNWs: Graphene
PECVD: Plasma-enhanced chemical vapor deposition
TS: Transparent and stretchable
Au: Gold
Ag: Silver
Cu: Copper
Pt: Platinum
Ni: Nickel
Al: Aluminum
MEMS: Micro-electro-mechanical system
Cr: Chromium
OTFT: Organic thin-film transistor
NPs: Nanoparticles
PE: Polyethylene
PTC: Positive temperature coefficient
VO_2_: Vanadium dioxide
PAD: Polymer-assisted deposition
NiO: Nickel oxide
P3HT: Poly(3-hexyl thiophene)
PPy: Polypyrrole
pNIPAM: Poly (N-isopropyl acrylamide)
SEBS: Polystyrene- block-poly(ethylene-ran-butylene)-block-polystyrene
PDPPFT4: Poly(diketopyrrolopyrrole-[3,2-b]thieno[2′,3′:4,5]thieno[2,3-d]thiophene])
PII2T: Poly(isoindigo-bithiophene)
OSCs: Organic semiconductors
TENG: Thermoelectric nanogenerator
DN: Double-network
DMSO: Dimethyl sulfoxide
P (VDF-TrFE): Copolymer polyvinylidene fluoride
IR: Infrared
FET: Field-effect transistor
BT: BaTiO_3_
ZnO: Zinc oxide
MFSOTE: Microstructure-frame-supported organic thermoelectric
PVD: Physical vapor deposition
CVD: Chemical vapor deposition
AlPcCl: Aluminum phthalocyanine chloride
RF-PECVD: Radio frequency plasma enhanced chemical vapor deposition
FCCVD: Floating catalyst chemical vapor deposition
PEI: Polyetherimide
LS: Laser writing
R2R: Roll-to-roll
EES: Electronic skin system
LDW: Laser direct writing
GNR: Graphene nanoribbon
tascPLA: Three-arm three-dimensional composite polylactide, polylactic acid
XRD: X-ray diffraction
m-LRS: Laser-induced reduction sintering
NTC: Negative temperature coefficient
ITO: Indium tin oxide
LEDs: Light emitting diodes
ECG: Electrocardiogram
DDP: Drug delivery pump
FWHS: Flexible wound healing system
FWTSD: Flexible wound temperature sensing device
UV: Ultraviolet
Si-NMs: Silicon nanomembranes
BES: Bioabsorbable electronic stent
HIFU: High-intensity focused ultrasound
AgNFs: Silver nanofibers
AgNWs: Silver nanowires
TLC: Thermochromic liquid crystal
RF: Radio frequency
